# Palaeoproteomic analysis of Pleistocene cave hyenas from east Asia

**DOI:** 10.1038/s41598-020-73542-x

**Published:** 2020-10-07

**Authors:** Huiyun Rao, Yimin Yang, Jinyi Liu, Michael V. Westbury, Chi Zhang, Qingfeng Shao

**Affiliations:** 1 Key Laboratory of Vertebrate Evolution and Human Origins, Institute of Vertebrate Paleontology and Paleoanthropology, Chinese Academy of Sciences, Beijing, 100044 China; 2 CAS Center for Excellence in Life and Paleoenvironment, Beijing, 100044 China; 3 State Key Laboratory of Palaeobiology and Stratigraphy, Nanjing Institute of Geology and Palaeontology, Chinese Academy of Sciences, Nanjing, 210008 China; 4 Department of Archaeology and Anthropology, University of Chinese Academy of Sciences, Beijing, 100049 China; 5 Section for Evolutionary Genomics, The GLOBE Institute, University of Copenhagen, Øster Farimagsgade 5–7, 1353 København K, Denmark; 6 College of Geographical Science, Nanjing Normal University, Nanjing, 210023 China

**Keywords:** Computational biology and bioinformatics, Evolution, Molecular biology

## Abstract

The spotted hyena (*Crocuta crocuta*) is the only extant species of the genus *Crocuta*, which once occupied a much wider range during the Pliocene and Pleistocene. However, its origin and evolutionary history is somewhat contentious due to discordances between morphological, nuclear, and mitochondrial data. Due to the limited molecular data from east Asian *Crocuta,* also known as cave hyena, and the difficulty of extracting ancient DNA from this area, here we present proteomic analysis of cave hyenas from three locations in northern China. This marks the first proteomic data generated from cave hyenas, adding new molecular data to the east Asian populations. Phylogenetic analysis based on these protein sequences reveals two different groups of cave hyenas in east Asia, one of which could not be distinguished from modern spotted hyenas from northern Africa, tentatively the result of previously suggested gene flow between these lineages. With developments of instrumentation and analytical methods, proteomics holds promising potential for molecular phylogenetic reconstructions of ancient fauna previously thought to be unreachable using ancient DNA.

## Introduction

Hyenas are feliform carnivoran mammals belonging to the family Hyaenidae, and originated in Early Miocene Eurasia (around 23–16 Ma). They reached the peak of their diversity during the Late Miocene (about 12–6 Ma), with approximately 100 fossil species found over Eurasia, Africa and North America^1,2^. However, since then, the distribution and diversity of hyenas have dramatically declined, with only four extant species remaining in Africa and southwest Asia (spotted hyena (*Crocuta crocuta*), striped hyena (*Hyaena hyaena*), brown hyena (*Parahyena brunnea*), and aardwolf (*Proteles cristatus*))^3^. The species that has gained the most systematic and evolutionary interest is the spotted hyena (*Crocuta crocuta*)^4,5^.

Although spotted hyenas are currently restricted to sub-Saharan Africa, this genus once inhabited almost the entire Eurasian continent during the Pliocene and Pleistocene, and comprised a variety of species, such as *Crocuta dietrichi*, *C. eturono*, *C. honanensis*, and the so-called Eurasian cave hyenas (which is further split into Asian and European lineages)^1,2,6^. As Eurasian cave hyenas are morphologically similar to extant spotted hyenas (*C. crocuta*), their taxonomic classifications have met contention. Cave hyenas have been considered either as a separate species from African spotted hyenas (*C. ultima and C. spelaea*) or as subspecies (*C. crocuta ultima* and *C. crocuta spelaea*)^1,7^. Furthermore, a recent study on the fossil hyena remains recovered from Geographical Society Cave in eastern Russia proposed the existence of two chronosubspecies, i.e. *C. ultima ultima* from the Middle Pleistocene of China and *C. u. ussurica* from the Late Pleistocene of the Russian Far East and China^8^. Moreover, the exact origins and evolutionary history of *Crocuta* have met with debate. Based on morphological analysis, it is unclear whether they originated in Africa or Asia and how they spread throughout Africa, Europe and Asia^9^.

**Figure 1:**
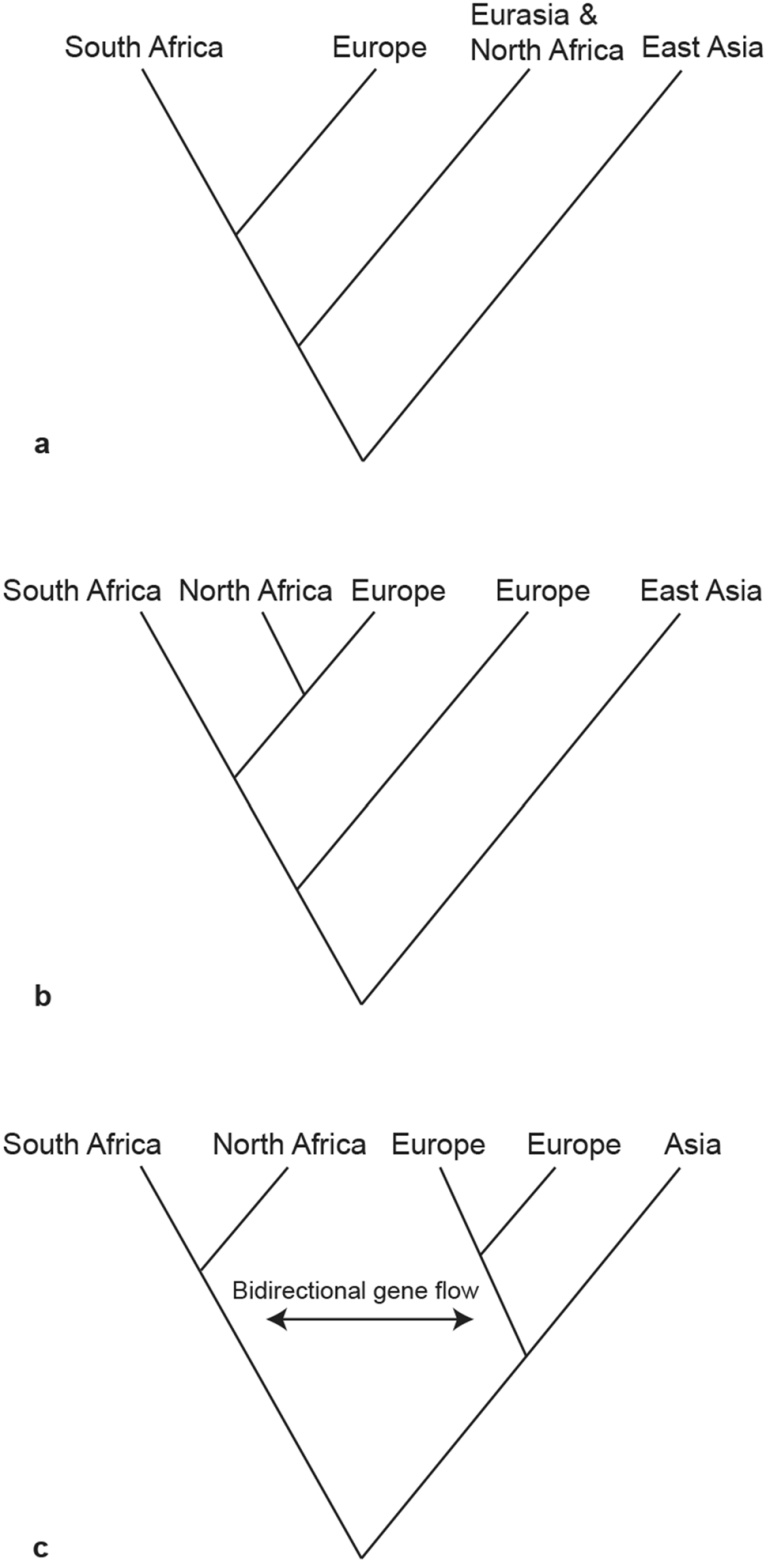
The phylogenetic relationships of modern spotted hyenas and cave hyenas based on different aDNA datasets: (**a**) short mitochondrial DNA fragments (366 bp of cyt b sequences)^10,11^, (**b**) complete mitochondrial genomes^12^, and (**c**) nuclear genomes^12^. All the analyzed samples from Africa were modern spotted hyenas and all the analyzed samples from Eurasia were Late Pleistocene cave hyenas. The figure was generated using Adobe Illustrator CC 2015 (https://www.adobe.com/cn/products/illustrator.html).

**Figure 2:**
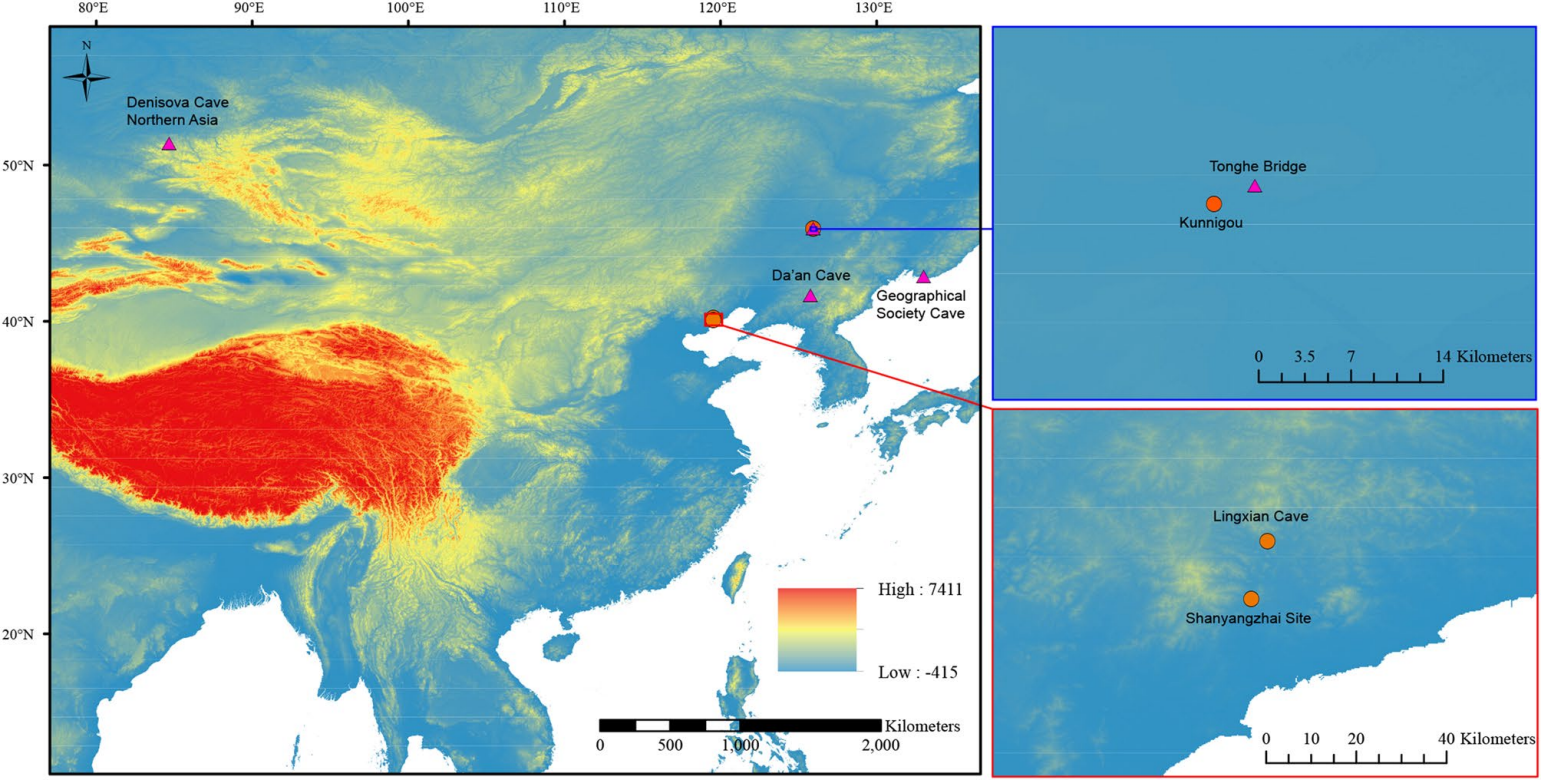
Geographical locations of cave hyenas with molecular data from Asia. The filled circles represent the three localities in this study that successfully provided proteomic data. The filled triangles represent the localities where genetic analyses have been successfully employed 10–12. Except Denisova Cave, located in northern Asia, all the other localities are in east Asia. Both Tonghe Bridge and Kunnigou are located in Zhaodong County, Heilongjiang Province. Both Lingxian Cave and Shanyangzhai Site are located in Qinhuangdao City, Hebei Province. The map was generated using ArcMap v10.2 (https://www.esri.com/software/arcgis/arcgis-for-desktop) on the basis of SRTM data courtesy of the U.S. Geological Survey (https://landsat.usgs.gov).

Ancient DNA has recently been applied to cave hyena subfossils to provide molecular insights in an attempt to resolve the issues mentioned above (Fig. 1). Several publications based on mitochondrial DNA revealed that western Eurasian cave hyenas are divided into two groups with one group intermingled with northern African spotted hyenas, while east Asian cave hyenas form a basal diverging lineage^10–12^. As to the origin and evolutionary history of spotted hyena fossils, Rohland et al.^10^ suggested three separate dispersal events out of Africa to Eurasia between 3.5 and 0.35 Ma, while Sheng et al.^11^ proposed an Eurasian origin at a far more recent evolutionary timescale (430–163 kya). These mitochondrial findings were later debunked by Westbury et al.^12^ who investigated the relationships between African spotted hyenas and Eurasian cave hyenas using ancient nuclear genomes. The nuclear genome results showed that spotted and cave hyenas form reciprocally monophyletic clades. When these results were interpreted together with the fossil record, they suggested an African origin followed by a dispersal into Eurasia shortly after 2.52 Ma. Moreover, they found evidence for several instances of bidirectional gene flow between the modern African populations and European cave hyenas^12^. However, due to the requirement of a putatively unadmixed comparative population in their analyses, it is unclear whether gene flow from Africa into Asia also occurred. The obscure evolutionary history of Asian populations could be caused by the limited number of samples analyzed so far (Fig. 2). Therefore, the question of whether there actually was gene flow between the Asian cave hyenas and other lineages is still unresolved. Furthermore, are there different lineages in east Asia, similar to what was uncovered in the European cave hyenas? To resolve these uncertainties, more molecular data is required from a wider sample set of east Asian individuals.

Recently, palaeoproteomic analyses have been successfully employed to resolve the phylogenetic relationships between extinct and extant mammalian taxa^13–17^. Moreover, ancient proteins are more resistant to diagenesis and could preserve longer than aDNA allowing the molecular study of extinct species past the reach of aDNA^18,19^. Collagen, which comprises the majority of the organic fraction of vertebrate bone and dentine, could not only be detected in mammalian fossils as old as mid-Pliocene (3.5 Ma)^20^, but also be recovered from tropical and subtropical areas^13,16^. Unlike bone/dentine which is an open system, enamel and eggshell have bigger mineral crystals which entrap proteins and behave as a closed system^21^. The degradation of these entrapped proteins is rarely influenced by other factors except time and temperature, which makes it possible to generate authentic proteins from warmer areas and from much older ages^22–24^. Previous phylogenetic analysis based on ancient protein sequences yielded highly congruent results with those based on aDNA, especially at high level classifications^13–15,25^. Therefore, palaeoproteomic analyses have great potential to provide further molecular data for phylogenetic reconstructions, especially when aDNA is not preserved.

Cave hyenas (*C. ultima*) survived in China from the Middle Pleistocene to the Early Holocene^26^. Dozens of fossil sites containing this species have been discovered, providing a massive amount of material for research^27^. However, some localities are not favorable for aDNA preservation, for example, aDNA failed to be extracted from samples of Lingxian Cave, Qinhuangdao City, Hebei Province, despite multiple attempts^11^. Here, we acquired, for the first time, palaeoproteomic data from cave hyenas of three localities in northern China (including Lingxian Cave). We performed phylogenetic analyses on the recovered protein sequences and provided further information to the evolutionary history of east Asian cave hyena populations.

## Studied localities and related samples

We collected four cave hyenas (two skulls and two mandibles with teeth) from three Pleistocene localities in northern China (Fig. 2 and Table 1). The two skulls were sent for radiocarbon dating at Beta Analytic Testing Laboratory. The sample from Kunnigou, Zhaodong County (coded as HZD) was AMS-dated greater than 43.5 kya (conventional radiocarbon age), but the sample from Shanyangzhai site (coded as SYZ) failed to be AMS-dated. Through faunal comparison, the chronology of Shanyangzhai site was inferred as Middle and Late Pleistocene^28,29^. The two mandibles with teeth (LXD-1 and LXD-2) were discovered from Lingxian Cave, which is also in Qinhuangdao City, 15 km away from the Shanyangzhai site^30^. Samples from both bone and dentine were taken from the mandibles and teeth respectively (abbreviated as LXD-1B, LXD-1D, LXD-2B, and LXD-2D). For both LXD-1 and LXD-2, three powdered samples were also drilled from the dentine for U-series dating using MC-ICPMS^31^ at Nanjing Normal University which yielded dates of 94.6 ± 0.2, 89.8 ± 0.2 and 92.5 ± 0.2 kya for LXD-1 and 99.2 ± 0.2, 99.0 ± 0.2 and 98.5 ± 0.2 kya for LXD-2. Given that the measured uranium was incorporated into the dentine after their burial; the U-series dates must be regarded as minimum age estimates for the corresponding mandibles^32^. To make our expressions clearer, hereafter we regard all the specimens from Lingxian Cave as one sample, and specific specimens like LXD-1B, LXD-1D, LXD-2B, and LXD-2D as its subsamples. After mechanically cleaning the exterior surface, the samples were ground into powder and divided into two aliquots, one used in the following analyses, and one to be stored as backup in the freezer at Key Laboratory of Vertebrate Evolution and Human Origins, IVPP for possible further analyses.

**Table 1:** The diagenetic parameters obtained by FTIR, elemental and ZooMS analyses and the coverage of type I collagen.

Sample code	Provenance	Age	Element	Am/P	IRSF	N wt%	C wt%	C:N ratio	α-value *	Coverage (%)^#^
HZD	Kunnigou, Zhaodong County, Heilongjiang Province	> 43.5 kya (AMS^14^C)	Skull	0.14	3.59	3.37	10.53	3.64	0.75	95.7
SYZ	Shanyangzhai site, Qinhuangdao City, Hebei Province	Middle and Late Pleistocene	Skull	0.03	5.30	0.52	5.10	11.37	0.19	89.8
LXD-1B	Lingxian Cave, Qinhuangdao City, Hebei Province	> 94.4 kya (U-series)	Mandible	0.02	4.74	0.18	5.11	32.53	0.06	79.6
LXD-1D	Lingxian Cave, Qinhuangdao City, Hebei Province	> 94.4 kya (U-series)	Tooth	0.02	4.76	0.13	3.86	33.93	n.d	
LXD-2B	Lingxian Cave, Qinhuangdao City, Hebei Province	> 99.0 kya (U-series)	Mandible	0.02	4.50	0.13	3.67	32.09	n.d	
LXD-2D	Lingxian Cave, Qinhuangdao City, Hebei Province	> 99.0 kya (U-series)	Tooth	0.02	4.83	0.26	4.32	19.72	n.d	

*The value (between 0.0 and 1.0) shows the level of undeamidated glutamine, with 0.0 being totally deamidated and 1.0 being not deamidated at all.

n.d. indicates that the α-value failed to be calculated due to poor signal of the peptide in the PMF spectrum.

^#^This value represents the coverage of type I collagen when the raw datafiles were searched against the custom carnivorous type I collagen database. It is higher than the coverages of COL1A1 and COL1A2 displayed in Table 2 because the signal peptides and propeptides were removed and these regions rarely survived in our fossil samples.

## Results and discussion

### Prescreening of the collagen preservation

ATR-FTIR (Attenuated total reflectance-Fourier transform infrared) analysis, elemental analysis, and ZooMS screening (Zooarchaeology by mass spectrometry) were employed to assess the preservation of collagen in the samples from three sites. The results of diagenetic parameters are displayed in Table 1. The infrared splitting factor (IRSF, defined in reference^33^), and the amide to phosphate ratio (Am/P, defined in reference^34^), were calculated based on the ATR spectra (Supplementary Figs. S9–14). The IRSF represents the crystalline structure of bones, and higher IRSF value indicates an increase of crystal size and order, which is related to the degradation of bone proteins^33,34^. After analyzing 195 bones excavated from 32 sites, Smith et al.^35^ suggested that extremely degraded/burnt bone would have IRSF values in excess of 4.0, which is in the case of samples from Shanyangzhai site (SYZ, hereafter represented as the SYZ sample) and Lingxian Cave (i.e. LXD-1B, LXD-1D, LXD-2B, and LXD-2D subsamples, here after referred to as the LXD sample). A lower IRSF value (3.59) of the sample from Kunnigou, Zhaodong County, Heilongjiang Province (HZD, hereafter represented as the HZD sample), indicates better preservation of collagen. The Am/P measures the relative content of collagen versus mineral apatite, and this parameter is confirmed to be positively correlated with the organic material or nitrogen content in the whole bone or dentine samples^34,36^. Several publications have reported the Am/P values of modern bones ranging from 0.16 to 0.42^36–38^. Chowdhury et al.^39^ also suggested that in combination with the position of the amide I peak, Am/P above 0.07 could be considered as an indicator of successful extraction of soluble collagen with ammonium bicarbonate protocol in ZooMS screening. In contrast to the HZD sample with a high Am/P value of 0.14, the SYZ sample had a low Am/P value of 0.03 while the LXD sample has an even lower average value of 0.02. The Am/P measurement validates the collagen preservation of the three sites predicted by IRSF.

The nitrogen content (N wt%) and the carbon:nitrogen atomic ratio (C:N ratio) of the whole bone are regularly used as indicators of collagen preservation before radiocarbon measurements^40^. According to Brock et al.^40^, the HZD sample has a N wt% above 3.0 and C:N ratio between 3.0 and 3.9, which suggests a probability of 100% and 92% respectively to successfully extract sufficient collagen for radiocarbon dating, i.e. > 1% of collagen yield^41^. This sample was successfully AMS-dated. The SYZ and LXD samples present much lower N wt% and higher C:N ratio, which was probably caused by the diagenesis effect in the following two mechanisms. Firstly, prolonged diagenesis leaded to the loss of collagen and the change of the amino acid composition, which in turn reduced the N wt% and increased the C:N ratio^42^. Moreover, the invasion of exogenous carbonate from groundwater or soil water after burial increased C wt% in the whole bone^43^. Similarly based on the criteria generated by Brock et al.^40^, the LXD sample has the worst collagen preservation, giving them little to no chance to be AMS-dated. The SYZ sample has a low probability to extract sufficient collagen, and we were unable to radiocarbon date this sample.

ZooMS screening reveals differences in the collagen spectral quality between samples. Of all the samples in the current study, the HZD and SYZ samples have higher quality of collagen spectra compared to the LXD sample (Supplementary Figs. S15–17). Except for the LXD-1B subsample, the other three LXD subsamples rarely displayed effective peaks. Based on the definition in reference^44^, we calculated the deamidation extent (represented as α-value) of a peptide biomarker at m/z 1105 to demonstrate the diagenetic alteration (P 1105, GVQGPPGPAGPR-hydroxylated at position 6 (underlined), common biomarker in terrestrial mammals^45^). Except for three of the LXD subsamples, whose α-values failed to be calculated due to poor signal of the peptide in the PMF spectrum, the results of the other three (sub)samples are presented in Table 1. The HZD sample has a lower deamidation level than the SYZ and LXD samples, which also suggests its better collagen preservation.

Summarizing all the prescreening results, it could be inferred that the HZD sample is well preserved with a good collagen signal. On the contrary, the SYZ and LXD samples (especially the LXD sample) have undergone more diagenetic alteration. However, there are still collagen signals with lower content. All the samples were loaded on the LC–MS/MS analysis with two enzyme digestions to more accurately perform protein identifications.

### Proteome composition and the authenticity of recovered proteins

Combining analyses from two enzymes (trypsin and elastase), we were able to generate high coverages of type I collagen sequences for the SYZ sample (89.8%), the HZD sample (95.7%) and the LXD sample (79.6%) (Table 1). The comparable results agree well with the collagen preservation estimated by the above prescreening analyses. It is noteworthy that a good coverage of sequence was still recovered from the LXD sample even though it presented extensive diagenesis. This shows the high preservation potential of collagen for phylogenetic analysis.

**Figure 3:**
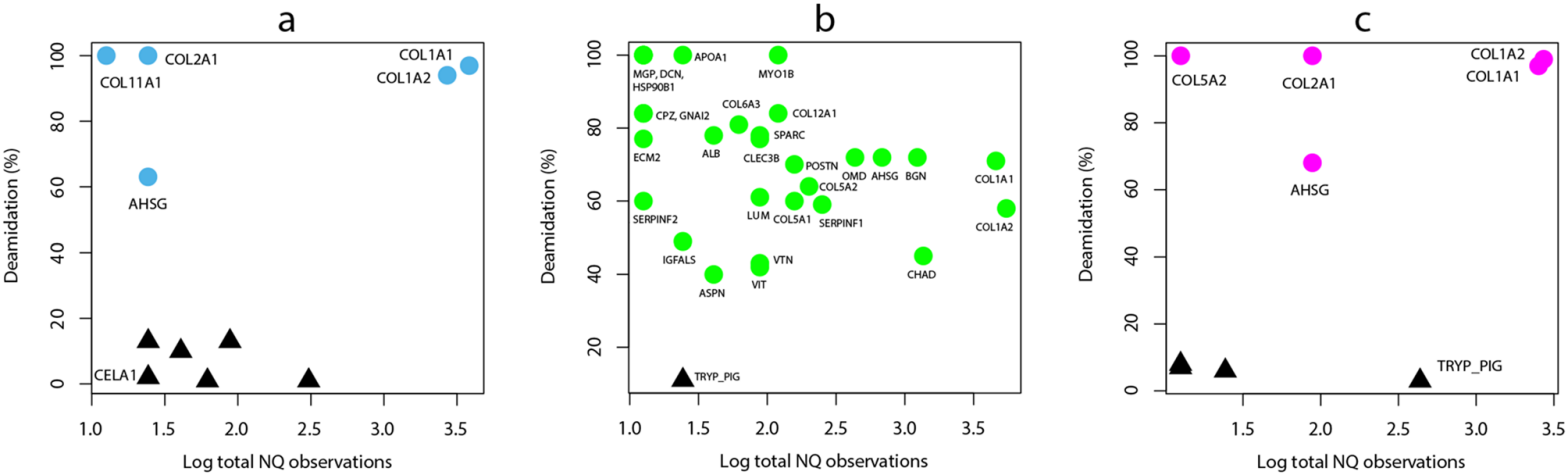
Deamidation frequencies of the identified Pleistocene proteins used in cluster analysis for the (**a**) SYZ, (**b**) HZD and (**c**) LXD sample. 0% indicates no deamidation and 100% indicates complete deamidation. Black triangles represent contaminating proteins, and circles represent endogenous proteins, with their gene names labelled beside. Note that some proteins have the same or close NQ observations and deamidation values, their circles or triangles are overlapped. The figure was generated using RStudio v1.2.1335 (https://rstudio.com/products/rstudio/).

In addition to type I collagen, other types of collagens and NCPs (non-collagenous proteins) were also detected in these samples (Fig. 3, Supplementary Tables S1–3). The extent of deamidation has recently been considered as a marker of diagenetic alteration and used to provide support for the authenticity of ancient sequences^46^. Here we calculated the deamidation frequencies for each protein quantitatively, based on the sum ion intensities of deamidated and undeamidated glutamine (Q)/asparagine (N) spectra. However, most of the spectra containing glutamine and asparagine positions in these proteins were not abundant enough for quantitative analysis. The deamidation frequencies could only be calculated for 26 proteins from the SYZ sample, 84 from the HZD sample and 27 from the LXD sample, among which the proteins suitable for cluster analysis (i.e. with more than two glutamine or asparagine positions covered) were even fewer. With group number set as 2, the cluster analysis results in a clear separation of endogenous and contaminating proteins (Fig. 3).

For the SYZ sample, only 12 proteins were chosen for cluster analysis. The first group of 7 proteins displays a low deamidation value (6.0% ± 5.7), including known protein contaminants (6 human skin keratins) and 1 added digestive enzyme (elastase, i.e. chymotrypsin-like elastase family member 1 (CELA1)), which comprise the exogenous protein group (Fig. 3a). In contrary, the other group of 5 proteins has obviously elevated deamidation frequencies ranging between 63 and 100%, including 4 collagens (COL1A1, COL1A2, COL2A1, COL11A1) and 1 NCP (AHSG), which have been reported to survive over the Early Pleistocene in paleontological and archaeological bone/dentine samples^19,47^. This group could be attributed as endogenous proteins. Among the other proteins which have none or only few (≤ 2) of the glutamine/asparagine positions covered by PSMs (peptide-spectrum matches), several proteins could be potentially considered as endogenous because of their endogeneity attribution in another sample of this study (the HZD sample), previous palaeoproteomic publications^15,48^, and the complete deamidation of the few glutamine/asparagine observations (COL3A1, COL4A3, COL4A4, COL5A1, COL5A2, COL9A1, COL16A1, POSTN, SERPINF1, etc.). The proteome composition and deamidation range of the LXD sample are similar to those of the SYZ sample (Fig. 3c).

For the HZD sample, the recovered proteome is more complex (Fig. 3b). The deamidation frequencies of 30 proteins were used in a cluster analysis. Except one protein (TRYP_PIG) with a low deamidation value (11%) which is one of the two digestive enzymes added during the protein extraction procedure, the other 29 proteins comprise a group with elevated deamidation values ranging between 40 and 100%. Bone proteins with different functions were detected in this group, including collagens (COL1A1, COL1A2, COL5A1, etc.), extra cellular matrix proteins related to bone (CHAD, CLEC3B, MGP, OMD, VTN, etc.), calcium (AHSG, ASPN, SPARC, etc.) and fibril (BGN, LUM, DCN, etc.), plasma protein (ALB), and intracellular protein (MYO1B). Most of these proteins are common bone/dentine proteins previously reported in ancient bone proteomes, and the elevated deamidation values confirm their endogeneity^48,49^. Similarly, some proteins excluded from cluster analysis, such as COL2A1, COL3A1, OGN, PROC, SPP2, could also be interpreted as endogenous to the analyzed bone sample^15,49,50^. The deamidation frequencies of COL1A1 and COL1A2 in the HZD sample were 71% and 58% respectively, which are significantly lower than those in the SYZ and LXD samples (over 90%) (Table 2). It indicates a decline in protein preservation for the SYZ and LXD samples, which is consistent with their decreased proteome complexity, dominated by type I collagen. This tendency has been reported previously^14,19^, which confirms an endogenous origin of the identified proteins in our study.

**Table 2:** The detailed information of the 10 proteins used to construct the phylogenetic datasets.

Proteins	Score	Coverage (%)	Peptides	Unique peptides	PSM	NQ count*	Deamidation
SYZ							
COL1A1	380.1	67	537	507	1915	36	0.97
COL1A2	322.88	60	347	343	991	31	0.94
AHSG	44.95	8	1	1	20	4	0.63
SERPINF1	41.46	9	2	2	5	1	1
HZD							
COL1A1	403.2	73	657	618	3618	39	0.71
COL1A2	384.74	71	478	471	1940	42	0.58
AHSG	255.03	62	89	15	388	17	0.72
BGN	193.34	49	40	40	178	22	0.72
CHAD	204.81	53	42	4	197	23	0.45
CLEC3B	109.38	45	7	7	13	7	0.77
OMD	191.16	33	32	14	166	14	0.72
SERPINF1	252.16	53	68	11	232	11	0.59
SPARC	156.16	35	22	6	63	7	0.78
VTN	179.56	32	38	16	107	7	0.43
LXD							
COL1A1	301.29	58	415	388	2745	30	0.97
COL1A2	280.94	53	257	255	1299	31	0.99
AHSG	106.94	20	11	11	107	7	0.68
SERPINF1	67.6	19	5	5	11	2	1
VTN	28.49	2	1	1	2	2	0.78

*This value represents the number of observed glutamines (Q) and asparagines (N) which have sufficient ion intensities for the deamidation calculation.

However, other than type I collagen, these additional proteins identified here have not been commonly used in phylogenetic analysis, partly because their sequence coverages are comparatively low compared to type I collagen. To obtain more complete sequences, 10 endogenous proteins with a coverage of over 30% in any of the three samples were chosen to generate the phylogenetic datasets (Table 2). Two datasets were constructed, one including only type I collagen (see Method, Supplementary Fasta File S1), and the other including 8 additional NCPs, i.e. AHSG, BGN, CHAD, CLEC3B, OMD, SERPINF1, SPARC and VTN (see Method, Supplementary Fasta File S2). The protein sequence sources of the comparative datasets are displayed in Supplementary Table S4, including accession numbers from Genbank/Uniprot and associated genome/proteome publications. It is notable that only the protein sequences translated from high coverage genomes were included in these datasets, as those translated from low coverage genomes (< 10x) were less reliable.

### Phylogenetic reconstruction

Our genomic/proteomic datasets are the most extensive protein sequence datasets available for Hyaenidae so far. Although type I collagen sequence for modern spotted hyena has once been reported in reference^48^, a reanalysis of their raw data against our proteomic database, resulted in a concatenated sequence without any amino acid substitutions compared to the sequences of modern spotted hyenas from northern Africa (see Supplementary information). However, the geographic location of this sample is unknown. Thus, this proteomic sample was excluded in our phylogenetic analysis.

**Figure 4:**
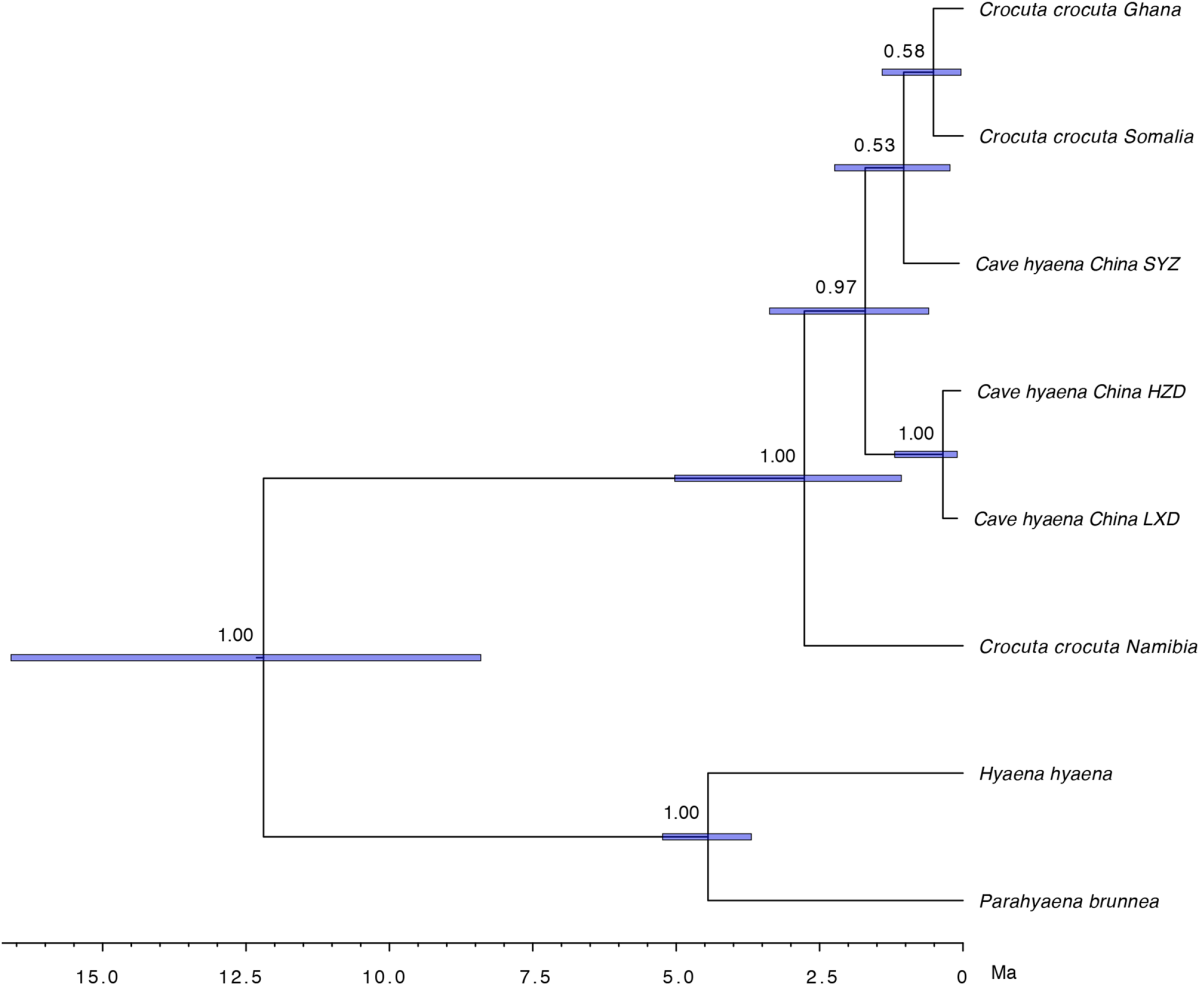
Time-scaled maximum clade credibility tree estimated using BEAST and a concatenated alignment of 10 proteins from 8 extant and extinct taxa. Posterior Bayesian probabilities are indicated at nodes with a probability of ≤ 1. Horizontal error bars at each node represent 95% highest posterior density intervals. The figure was generated using FigTree v1.4.3 (https://tree.bio.ed.ac.uk/software/figtree/).

We built several phylogenetic trees using parsimony and Bayesian methods based on two datasets, which result in largely concordant topologies within the Hyaenidae clade (Fig. 4, Supplementary Figs. S3 and S4). In these trees, the Namibian individual forms a clade basal to the other modern and fossil spotted hyenas, where the cave hyenas from China are divided into two clades. HZD and LXD form a monophyletic clade while SYZ groups with modern spotted hyenas from northern Africa (Ghana and Somalia). The phylogenies based on protein data are discordant with the results generated from nuclear genomes^12^, where cave hyenas from Eurasia and modern spotted hyenas from Africa form reciprocal monophyletic clades. The discordance between nuclear and mitochondrial phylogenies was interpreted as putative gene flow^12^, which could also fit the case of east Asia here. The phylogenies constructed from both of our proteomic datasets indicate the Namibian individual as a basal lineage, which agrees with the result of gene flow analysis, i.e. the Namibian individual contained the fewest windows of gene flow from cave hyenas compared with other modern individuals^12^. Moreover, considering the amino acid substitutions in the *Crocuta* populations (see Supplementary Information), the Namibian individual represented the ancestral type of amino acid (Isoleucine, i.e. I) at position 1449 (COL1A2, the position number was derived from the dataset alignments) while all the other modern and fossil spotted hyenas displayed the derived type (Valine, i.e. V), with high depth of PSM sequence coverage in the three fossil samples. Additional phylogenetic analyses without this variable site (all the Hyaenidae individuals called as X at position 1449) result in different topologies (see Supplementary Information, Supplementary Figs. S5 and S6), especially the placement of the Namibian individual. It indicates the significant importance of this specific variable site for the gene flow deduction in our study. However, it is noteworthy that the frequency distribution of different amino acid types (I, V, possibly other types detected afterwards at position 1449) within Hyaenidae populations remains unclear yet due to limited number of whole genomes available so far. With more samples analyzed in the future, the phylogenetic relationships between modern spotted hyenas and east Asian cave hyenas could be confirmed or modified.

The Bayesian analysis carried out in BEAST indicates that all the analyzed spotted hyenas, including modern individuals from northern and southern Africa and fossils from east Asia, share the most recent common ancestor (tMRCA) at ~ 2.77 Ma, which agrees well with the time estimated from nuclear genomes^12^. Furthermore, based on the divergence time between the SYZ and the northern African individuals (Fig. 4), we suggested the putative gene flow could occur sometime before ~ 1.03 Ma (95% highest posterior density interval, 0.23–2.24 Ma). Combining the genome data with the oldest *Crocuta* fossil records in Africa (*Crocuta dietrichi*, 3.63–3.85 Ma)^51^, east Asia (*Crocuta honanensis*, ~ 2 Ma)^6^ and Europe (~ 0.9 Ma)^52^, recently Westbury et al.^12^ suggested a dispersal from Africa into Eurasia, most likely into Asia, and then followed by subsequent dispersal into Europe from Asia. The estimated time of putative gene flow between northern African and east Asian populations coincides with the dispersal from Asia into Europe. However, more evidence is needed to accurately determine whether this gene flow had any further impact on the European cave hyenas.

Here in our study, we presented the first ancient protein sequences from cave hyenas, providing more molecular data from new localities and time bins in east Asia. Two different groups were found in the east Asian populations, partly verifying the two subspecies statement proposed by Baryshnikov based on morphological analyses^8^. Even though both the SYZ and LXD cave hyenas were discovered in Qinhuangdao City, they belong to different groups, which questions the geographic correspondence of cave hyenas. A similar phenomenon could also be observed in the case of European cave hyenas where the cave hyenas could be separated into two groups without any obvious temporal or geographic correspondence^10–12^. Endogenous proteins were successfully extracted from the locality unfavorable for aDNA preservation (for example, Lingxian Cave), which demonstrates the potential of palaeoproteomic analysis in phylogenetic reconstructions when DNA cannot be recovered. It could be employed to resolve more phylogenetic issues in the future, especially the placement of other species that went extinct much earlier in time, such as *Crocuta honanensis,* and *Pachycrocuta*.

## Conclusion

In conclusion, multiple approaches were applied to cave hyena samples from three localities in northern China. Combining the prescreening and proteomic analyses, endogenous proteins have been identified, especially for the HZD sample, which is well preserved and displays a complex bone proteome. Using the recovered collagen and NCP sequences, phylogenetic reconstructions suggest a putative gene flow between northern African and east Asian populations occurred sometime before ~ 1.03 Ma and that there may have been two different groups of east Asian cave hyenas. Furthermore, our data provides new and fundamental reference data for future comparisons. With the addition of more samples and more sources of data for phylogenetic reconstructions, we foresee that the evolutionary history of cave hyenas will be revealed in the future.

## Methods

### ATR-FTIR analysis

Attenuated total reflectance-Fourier transform infrared (ATR-FTIR) analysis was employed to assess the preservation of collagen using a Nicolet 6700 (Thermo Scientific) FTIR spectrometer. The ATR crystal of the spectrometer was pressed directly on the bone or dentine powder, without any additional sample treatment. The background was collected before each analysis. Spectra were acquired over the range of 4000–525 cm^−1^ with a resolution of 4 cm^−1^ and 32 scans per spectrum. The software OMNIC v8.0 (https://www.thermofisher.com/order/catalog/product/INQSOF018#/INQSOF018) was applied to analyze the data.

### Elemental analysis

Approximately 1 mg of bone or dentine powder was weighed and packed into a tin capsule for combustion. Carbon (C wt%) and nitrogen (N wt%) contents were measured by a CHNS-O Analyzer (Flash 2000, Thermo Scientific).

### Protein extraction and analysis

#### Protein extraction

The extraction procedure was modified from references^53,54^. In brief, approximately 200 mg of bone or dentine powder was demineralized in 0.6 M HCl. After demineralization and centrifugation, the acid supernatant was removed. The acid-insoluble residue was incubated in 200 μl 50 mM ammonium bicarbonate buffer at 65 ℃ for 3 h. After centrifugation, 100 μl of the supernatant was digested with 3 μl of 0.5 μg μl^−1^ porcine trypsin (Promega) overnight at 37 °C. The other 100 μl was dried and resuspended in 100 μl 10 mM Tris HCl solution (PH = 8), and digested with 4 μl of 1 μg μl^−1^ elastase (Worthington) overnight at 37 °C. Digestion was stopped by adding trifluoroacetic acid (TFA) to a final concentration of 0.1%. Pierce C18 Pipette Tips (Thermo Scientific) were used for the desalting and elution of peptides. A small aliquot of the trypsin digest was used for ZooMS screening. All the other trypsin and elastase digests were dried up for the LC MS/MS analysis respectively. Multiple enzyme digests were used to increase the overall sequence coverage of identified proteins.

#### ZooMS

3 μl of trypsin digest and 3 μl of α-cyano-4-hydroxycinnamic acid matrix solution (1% in 50% ACN/0.1% TFA (v/v/v)) were mixed together and then spotted in triplicate onto an MTP384 Bruker ground-steel MALDI target plate. After drying, all the spots were analyzed using a Bruker Autoflex Speed MALDI-TOF/TOF mass spectrometer (Bruker Daltonics) equipped with a 2000 Hz solid-state Smartbeam Nd:YAG UV laser (355 nm, Azura Laser AG). Mass spectra were acquired over a range of m/z 600 to 3500. Each spectrum was recorded from an accumulation of 20 laser scans, with each scan accumulated from 500 laser shots. A mixture of peptides was used for external calibration (peptide calibration standard II [Bruker Daltonics]: bradykinin 1–7 ([M + H]+, m/z 757.3992), angiotensin II ([M + H]+, m/z 1046.5418), angiotensin I ([M + H]+, m/z 1296.6848), substance P ([M + H]+, m/z 1347.7354), bombesin ([M + H]+, m/z 1619.8223), renin substrate ([M + H]+, m/z 1758.9326), ACTH clip 1–17 ([M + H]+, m/z 2093.0862), ACTH clip 18–39 ([M + H]+, m/z 2465.1983), and somatostatin 28 ([M + H]+, m/z 3147.4710)). After conversion of the raw files into text files using flexanalysis 3.4 (Bruker), the spectra were processed with the open-source cross-platform software mMass v5.5.0 (www.mmass.org)^55^. MALDI-TOF–MS replicates (n = 3) were averaged for the same sample. Peaks were picked with a S/N threshold of 3.5 and a relative intensity threshold of 0.5% after baseline suppression, smoothing and deisotoping were applied. The commonly used peptide markers (P-G)^48,53^ were manually inspected. In addition, the deamidation extent (represented as α-value) of a peptide biomarker at m/z 1105 was calculated from a weighted average of the three replicates of each sample. The details are descripted in reference^44^ and the code is available as an R package from GitHub (https://github.com/bioarch-sjh/q2e).

#### LC–MS/MS

The tandem mass spectrometry analysis was modified from reference^56^. In brief, the sample re-dissolved in water was analyzed on an on-line Q Exactive mass spectrometer coupled to an EASY-nano-LC 1200 system (Thermo Fisher Scientific, MA, USA). 1.5 μL of peptide was loaded on a trap column (Thermo Fisher Scientific Acclaim PepMap C18, 100 μm × 2 cm) and an analytical column (Acclaim PepMap C18, 75 μm × 15 cm). The samples were separated with a 60 min linear gradient, from 5% B (B: 0.1% formic acid in ACN) to 35% B. The column flow rate was set as 300 nL/min at 40 °C. The electrospray voltage of 2 kV versus the inlet of the mass spectrometer was used. The mass spectrometer was run under data dependent acquisition mode, and automatically switched between MS and MS/MS mode. The full scan range was set between 300 and 1800 m/z with a resolution of 70,000, an AGC target of 3e6, a maximum injection time of 60 ms and a dynamic exclusion time of 10 s. The MS/MS scan was performed at 17,500 resolution with an AGC target of 5e4, a maximum injection time of 80 ms and a collision energy of 27.

#### Protein sequence analysis

MS/MS datafiles from the same locality, including trypsin and elastase digestions, were merged and searched using PEAKS X (https://www.bioinfor.com/peaks-studio/) against a custom carnivorous type I collagen database. The database was generated from Genbank, UniProt, and specific publications, among which modern hyena sequences were either recovered from previous MS/MS analysis or predicted from genomes^12,48,57^. To keep the uniformity of the collagen entries, the COL1A1 and COL1A2 sequences were concatenated and signal peptides and propeptides were removed as these regions rarely survive in fossils. Common contaminants (https://www.thegpm.org/crap/) were also included in the database search. PEAKS searches, including Peptide de novo, PEAKS DB, and SPIDER, were performed with a fragment ion mass tolerance of 0.05 Da and a parent ion tolerance of 7 ppm, in addition to respective enzyme details (trypsin digestions set to semiTrypsin and elastase digestions set to NONE). A maximum of two missed cleavages were permitted. Searches allowed for up to six modifications per peptide, with oxidation (M), hydroxylation (P), hydroxylation (K), and deamidation (NQ) specified as variable modifications. False discovery rate (FDR) was set at 0.5% and protein scores were filtered with − 10lgP ≥ 20 and ALC (%) ≥ 50 (de novo only). Each locality (represented as HZD, SYZ, and LXD, Table 1) generated a potential consensus type I collagen sequence. The sequence was added into the custom collagen database, and the original data were merged and re-analyzed using PEAKS X against the new database to confirm the sequence coverage and substitutions. The authenticity of substitutions was inspected manually based on the criteria established by Presslee et al.^16^ and Hendy et al.^50^, i.e. a minimum of two peptide-spectrum matches (PSMs), the presence of both b and y ions, and the position of hydroxyproline (for choosing between hydroxyproline-alanine and proline-serine). If it was uncertain to confirm the presence of a possible substitution, the site was called as ‘X’ (missing sequence).

As no annotated complete genome of the Hyaenidae species was available online at the time, a homologous proteome from domestic cat (*Felis catus*; GenBank assembly accession: GCA_000181335; number of protein sequences = 32,891) was downloaded from Uniprot as a reference database to obtain the bone and dentine proteomes of these Pleistocene samples. The raw data were run against the entire domestic cat proteome database with the addition of a selected group of common bone and dentine proteins from Carnivora, especially the family Hyaenidae, which were manually translated from published genomes^12,57^. Common contaminants were included in the database as well. The parameters were set the same with those of the collagen database search in PEAKS X. Protein matches were accepted with − 10lgP ≥ 20 and one unique peptide. PTM profile results could provide a direct summary of the quantitative information (e.g. abundance of modified and unmodified forms covering the PTM sites). The deamidation frequencies of each identified protein were calculated based on summed ion intensities of the (non)deamidated peptides to discriminate endogenous from contaminating proteins. For each sample, the deamidation frequencies of proteins with more than two glutamine and/or asparagine positions covered by PSMs were imported into R Studio. Cluster analysis (R package Mclust) was applied to assign group membership using the deamidation frequency data^48^. Group number was set to 2 and other parameters remained as default settings.

### Phylogenetic analysis

Two comparative datasets were constructed for phylogenetic analyses. Dataset 1 was built using consensus sequences for COL1A1 and COL1A2 obtained by MS/MS analysis of the cave hyenas from three Pleistocene localities as well as the available type I collagen sequences of extant Feliformia species from Genbank/Uniprot and associated genome/proteome publications (Supplementary Table S4). *Ailuropoda melanoleuca* (giant panda) was set as an outgroup. Dataset 2 included the sequences of 8 endogenous NCPs (non-collagenous proteins) as well as COL1A1 and COL1A2. The NCPs were included when their sequence coverages were above 30% in at least one of the fossil samples (alpha-2-HS-glycoprotein [AHSG], biglycan [BGN], chondroadherin [CHAD], tetranectin [CLEC3B], osteomodulin [OMD], pigment epithelium-derived factor [SERPINF1], osteonectin [SPARC], and vitronectin [VTN]). This dataset comprised 9 entries: three modern spotted hyenas (*Crocuta crocuta*) from Africa, three cave hyenas from China, one striped hyena (*Hyaena hyaena*), one brown hyena (*Parahyena brunnea*), and one domestic cat (*Felis catus*, as an outgroup). Referencing database sources (with accession numbers) or associated genome publications are displayed in Supplementary Table S4. The sequences were concatenated and the signal peptides and propeptides of COL1A1 and COL1A2 were removed. For dataset 1, COL1A1 ranged from position 1 to position 1057, and COL1A2 ranged from 1058 to 2098. For dataset 2, the ten proteins were concatenated in the following order: COL1A1 = 1–1057; COL1A2 = 1058–2098; AHSG = 2099–2462; CHAD = 2463–2821; OMD = 2822–3237; SPARC = 3238–3540; VTN = 3541–4012; BGN = 4013–4384; CLEC3B = 4385–4586; SERPINF1 = 4587–5003. Leucines (L) were converted into isoleucines (I) as they are isobaric and cannot be discriminated by low energy tandem mass spectrometry.

Sequence alignments were performed in MEGA X using the MUSCLE algorithm with default settings, and then checked manually^58^. Dataset 1 comprised a total alignment length of 2,098 amino acid positions and dataset 2 included 5,003 amino acid positions. Three methods were performed on the aligned datasets to verify the phylogenetic results. Parsimony analysis was conducted through PAUP* version 4.0a (build 167)^59^, using a heuristic search with 1000 bootstrap replicates, and generating a 50% majority rule consensus tree. Two sets of phylogenetic analyses were performed in a Bayesian framework. We first ran PartitionFinder v.2.1.1^60–62^ to select the most appropriate partitioning schemes and substitution models for our datasets, which were used in the further phylogenetic analysis. The first set of Bayesian phylogenetic analysis used MrBayes v.3.2.6^63^. Two Markov chain Monte Carlo (MCMC) runs were performed. Two million generations were run with sampling every 1,000 generations. Convergence was checked based on the average standard deviation of split frequencies and the log likelihood values (LnL). Effective sample sizes (ESS) were assessed in TRACER v.1.6^64^. After a 40% burn-in, all parameters provided an ESS value of greater than 200, showing sufficient sampling. Summarizing the remaining samples, a 50% majority rule consensus tree was constructed with clade frequencies interpreted as posterior probabilities.

The other set of Bayesian phylogenetic analysis was performed in BEAST v.2.6.2^65^ to obtain a time calibrated tree for the ten proteins used in dataset 2. For this analysis, *Felis catus* was excluded and only 8 Hyaenidae entries were used. The partitioning schemes and substitution models referred to those used in MrBayes, but a WAG model was used to replace VT model due to the absence of the VT model in BEAST2^65^. Three ancient samples were calibrated using their mean ages, that is, SYZ at 69 ka (the median age of the Late Pleistocene range^28,29^), HZD at 43.5 ka and LXD at 99.2 ka. Moreover, we also enforced two node calibrations derived from previous studies^12,66–68^. The root age was assigned to 11.2 ± 2.5 Ma (gamma distribution)^12^, and the *Hyaena/Parahyaena* divergence date was assigned to 4.625 ± 0.4 Ma (normal distribution)^66–68^. As the samples are closely related species (except *Hyaena hyaena* and *Parahyaena brunnea* which are two genera forming a sister clade of the focal species), we assumed a strict molecular clock^69^ and a coalescent model with constant population size^70,71^ in the analysis. The clock rate was given a uniform (0,1) prior and population size was given a 1/X prior, which are default settings in the program. The MCMC chain was run for 10 million generations and sampled every 100 generations. The first 20% of samples were discarded as burn-in and the remaining samples were used to summarize the tree and parameter estimates. The convergence of the MCMC was examined using TRACER v.1.6^64^ to make sure that the effective sample size (ESS) of each parameter was larger than 200 and two independent runs produced consistent estimates. The maximum clade credibility (MCC) tree was summarized using TreeAnnotator included in the BEAST2 program.

## Supplementary information

Supplementary Information

Fasta File S1

Fasta File S2

XML File S1

XML File S2

XML File S3

NEX File S1

NEX File S2

NEX File S3

NEX File S4

Extended Data File S1

Supplementary Tables

## Data Availability

The mass spectrometry proteomics data have been deposited to the ProteomeXchange Consortium via the PRIDE partner repository with the dataset identifier PXD020530 and 10.6019/PXD020530. The authors declare that all data supporting the findings of this study are available within the paper and its supplementary materials.
