# Supplementary material for: Palaeoproteomic analysis of Pleistocene cave hyenas from east Asia
Source: Sci Rep. 2020 Oct 7;10:16674. doi: 10.1038/s41598-020-73542-x (PMC7541484; doi:10.1038/s41598-020-73542-x)
Supplement: Supplementary file 1 — Supplementary Information [file 41598_2020_73542_MOESM1_ESM.pdf]

Supplementary information for

Palaeoproteomic analysis of Pleistocene cave hyenas from east Asia

Huiyun Rao, Yimin Yang, Jinyi Liu, Michael V. Westbury, Chi Zhang, Qingfeng Shao

Crucial amino acid substitutions in cave hyenas

Of the 2,098 amino acids in dataset 1 (the type I collagen dataset with 15 Feliformia taxa and giant panda as an outgroup), 53 (2.5%) were variable and 28 (52.8% of variable sites, 1.3% of total) were parsimony informative for the represented taxa. By comparison, of the 5,003 amino acids in dataset 2 (the proteome dataset with eight Hyaenidae taxa and domestic cat as an outgroup), 201 (4.0%) were variable and 25 (12.4% of variable sites, 0.5% of total) were parsimony informative for the taxa represented. Even within a lower rank, the dataset 2 has a higher percentage of variable sites, which agrees with the higher sequence variability of NCPs than type I collagen [1,2]. However, the percentage of parsimony informative sites in dataset 2 was very low. After checking all the variable sites in dataset 2, it was discovered that 82.1% (165/201) of the variable sites were domestic cat specific while the remaining 36 sites were variable within the eight Hyaenidae taxa.

When restricted to the Hyaenidae taxa, 13 variable sites were present along the type I collagen sequences, among which one was specific for striped hyena, two for brown hyena, and seven for striped and brown hyena. One crucial variable site was detected at position 1449 (I>V, COL1A2, hereafter the position number was derived from the dataset alignments), which contains two types of amino acids. Isoleucine (I) was displayed in striped hyena, brown hyena and the Namibian individual (southern Africa), which may be an ancestral type. Valine (V) was detected in the other two modern spotted hyena individuals from Somalia (northeast Africa) and Ghana (northwest Africa), which we regard as the derived type. For the three cave hyena sequences, this variable site was all called as Valine (V) with a coverage of 183 (HZD), 83 (SYZ), and 69 (LXD) PSMs respectively, which is consistent with the modern spotted hyena sequences from northern Africa. Examples of the PSMs covering this region are showed in Fig. S1.

For the cave hyena sequence from the SYZ sample, a specific amino acid substitution was detected (H>D, COL1A2 at position 2049), with a coverage of 14 PSMs. One of the MS/MS product ion spectra for peptide TGDPGSVGPAGVR (2047-2059) from the SYZ sample is displayed in Fig. S2a, where the amino acid substitution (D) was identified by both y and b ions. However, the deamidation of asparagine (N) could result in the inference of aspartic acid (D). The average deamidation frequency for all glutamine/asparagine positions (n = 31) in COL1A2 from the SYZ sample was as high as 0.94, and the average deamidation frequency for asparagine (N) positions (n = 18) only was higher (0.97). We could not exclude the possibility that the amino acid call at position 2049 is a full deamidated asparagine (N). To confirm the original call of this position, we searched the carnivoran type I collagen database, where this specific position was a variable site with two types of amino acids, i.e. histidine (H) and glutamine (Q). When restricted to the represented Feliformia taxa, it was a conserved site with the same amino

acid call, i.e. histidine (H). Neither asparagine (N) and aspartic acid (D) were observed at this position, but a previous study has reported a H>D substitution in COL1A2 from a Pleistocene *Equus* sp. sequence [3]. In addition, the short peptide “TGDP” could be found in most of the represented carnivoran taxa at another region (1480-1483) while “TGNP” could not be detected. Thus, we consider this amino acid substitution as D rather than N.

For the cave hyena sequences from the HZD and LXD samples, another specific amino acid substitution was detected (E>S, COL1A1 at position 523), with a coverage of 9 (HZD) and 33 (LXD) PSMs respectively. Examples of the MS/MS product ion spectra for peptide GFPGSRGVQGPPGPAGPR (519-536) from the HZD and LXD samples are displayed in Fig. S2b and c. Besides type I collagen, two amino acid substitutions were detected in the HZD sample (K>R, CHAD at position 2561, and F>I, OMD at position 3175), both with a coverage of only 2 PSMs, which may require confirmation when more sequences become available for the carnivoran taxa in the future.

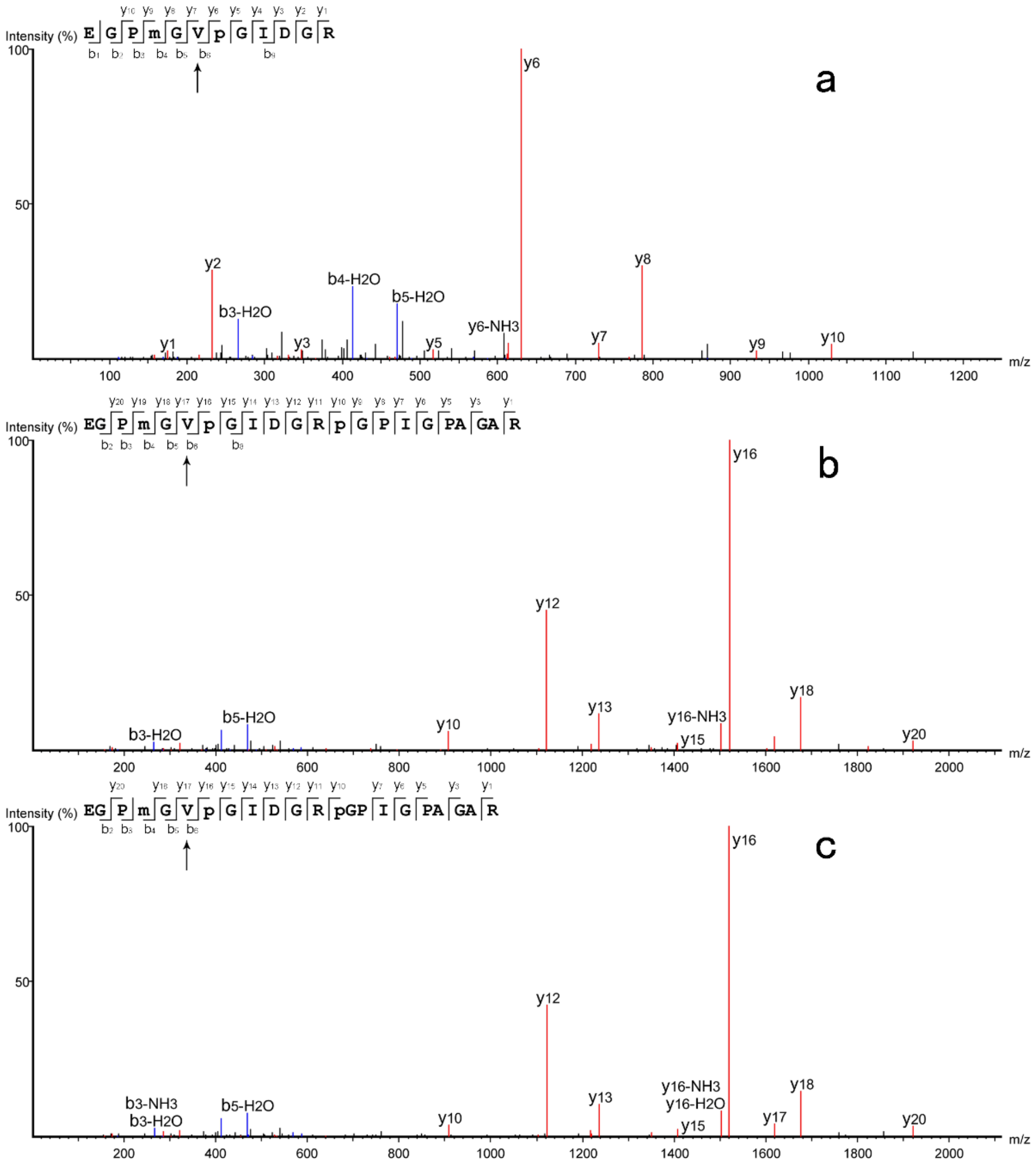

Figure S1. Examples of MS/MS product ion spectra covering the variable site at position 1449 (I>V, COL1A2) from the a) SYZ, b) HZD and c) LXD samples. Both oxidation (M) and hydroxylation (P) were detected and presented as lowercase letters. Arrows point out the positions of the variable site. The ion match tables for the spectra are displayed below (Figs. S18-20). The spectra were exported from PEAKS X (https://www.bioinfor.com/peaks-studio/). The arrows and the y/b numbers were added to the Roepstorff nomenclature diagrams using Adobe Photoshop CC 2015 (https://www.adobe.com/cn/products/photoshop.html).

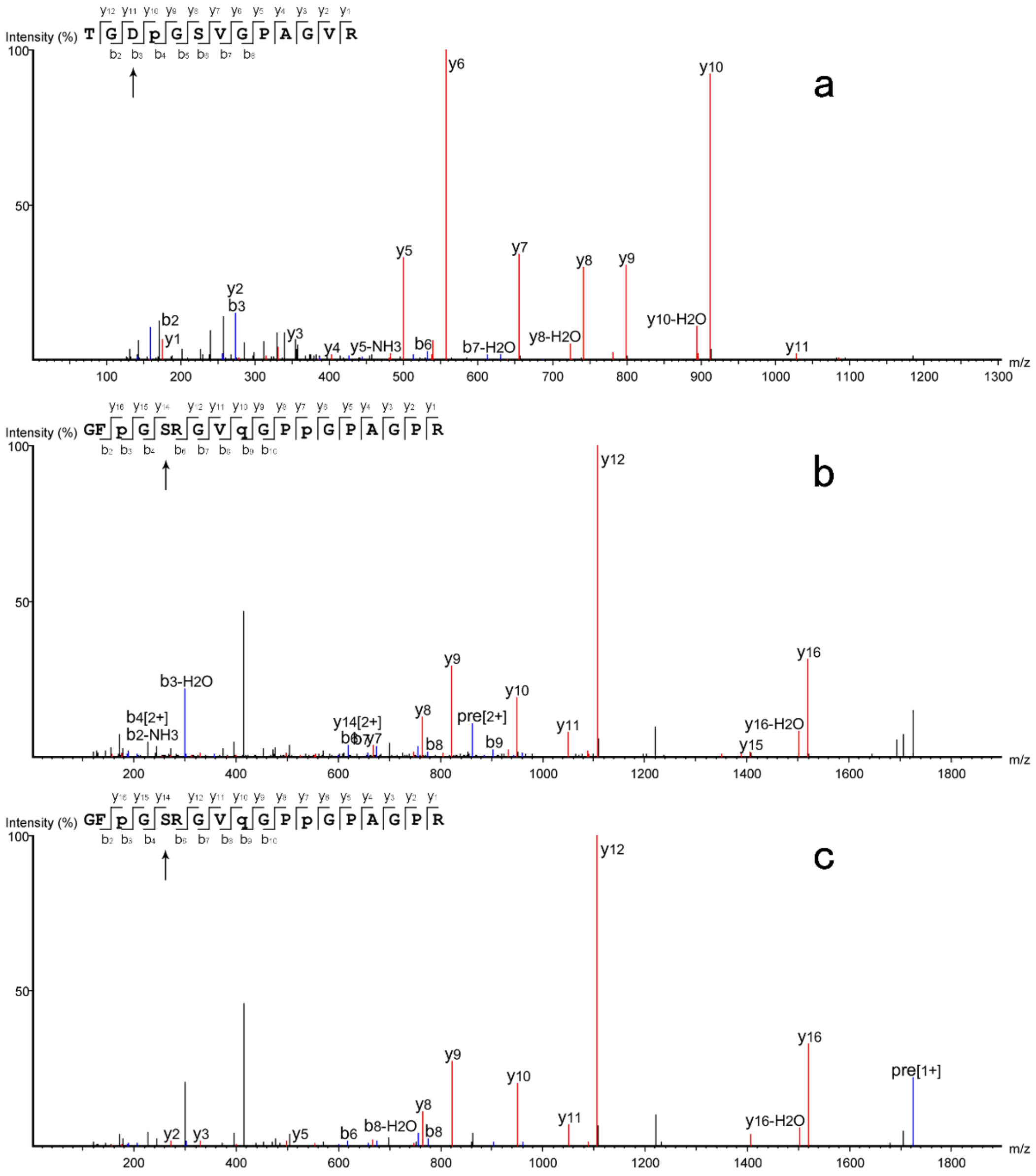

Figure S2. Examples of MS/MS product ion spectra covering the specific amino acid substitutions. a) MS/MS product ion spectrum covering the amino acid substitution (H>D, COL1A2 at position 2049) from the SYZ sample. b) and c) MS/MS product ion spectra covering the amino acid substitution (E>S, COL1A1 at position 523) from the HZD (b) and LXD samples (c). Both deamidation (Q) and hydroxylation (P) were detected and

presented as lowercase letters. Arrows point out the positions of the variable sites. The ion match tables for the spectra are displayed below (Figs. S21-23). The spectra were exported from PEAKS X (https://www.bioinfor.com/peaks-studio/). The arrows and the y/b numbers were added to the Roepstorff nomenclature diagrams using Adobe Photoshop CC 2015 (https://www.adobe.com/cn/products/photoshop.html).

Reanalysis of previously published MS/MS raw data of a modern spotted hyena

The type I collagen sequence for modern spotted hyena has once been reported in reference [4], where the MS/MS datafile was searched against a mammalian collagen database without any Hyaenidae sequence included. In that database, the closest relative of hyenas was from the family Felidae, which also belongs to the suborder Feliformia. In contrast, the local database in our study contained sequences from three modern spotted hyenas (*Crocuta crocuta*) from Africa, one striped hyena (*Hyaena hyaena*) and one brown hyena (*Parahyena brunnea*), which were all generated from high coverage genomes [5,6]. The raw data from reference [4] was downloaded and re-analyzed here with our new database and the same parameters described in the methods. The newly generated sequence has a greater coverage than previously reported (92.7% vs. 88.9%), and there are nine amino acid differences between the two *Crocuta* sequences, which is a substantial number considering the highly conserved nature of type I collagen. For seven of the nine amino acid differences, our newly developed sequence presented the same amino acid call as the Hyaenidae reference sequences while the sequence previously reported gave the same amino acid call as the Felidae reference sequences. Thus, most of the amino acid differences would be attributed to the improved database, whose effect has been discussed before [7,8]. The newly developed sequence for the modern sample is considered more reliable than that from reference [4]. For this sample, no reliable amino acid substitution was detected compared to the sequences of modern spotted hyenas from northern Africa. However, the geographic location of this sample is unknown. Therefore, its sequence was excluded from the phylogenetic datasets.

Phylogenetic reconstructions based on parsimony and Bayesian analyses (carried out in MrBayes) (dataset 1 and 2)

Parsimony and Bayesian analyses (carried out in MrBayes) result in largely concordant phylogenetic trees. Especially for the Hyaenidae clade, both datasets generate the same topology from two different methods (Figs. S3 and S4). The lower bootstrap support (BS) under parsimony analysis is possibly due to too few parsimony informative sites of the datasets, while Bayesian analysis makes full use of the data and results in higher posterior probabilities (PP). The support difference of two phylogenetic methods has been reported in a previous publication [7].

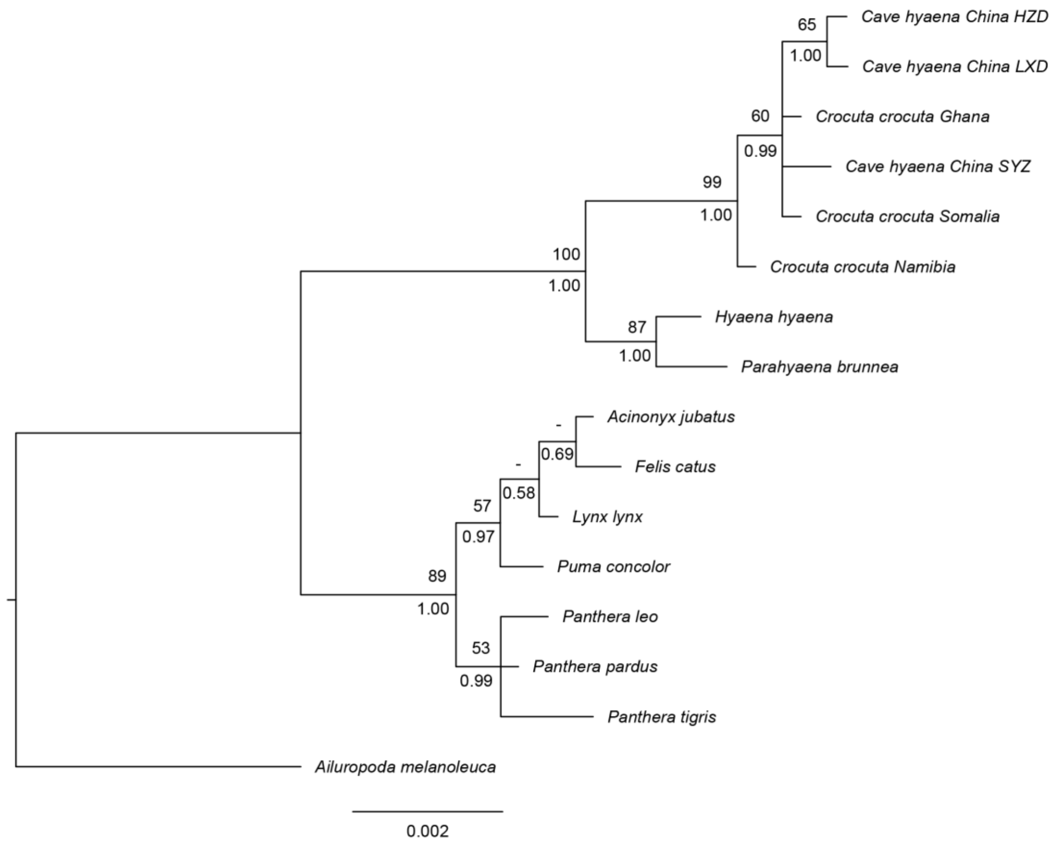

Figure S3. Fifty percent majority rule Bayesian consensus tree based on proteomic dataset 1, with *Ailuropoda melanoleuca* (giant panda) as an outgroup. Scale bar represents branch length in units of substitutions per site. Values below nodes are Bayesian posterior probabilities for the descendant clade. The parsimony results are also attached on the consensus tree, with values above nodes indicating the percent bootstrap support generated from 1,000 bootstrap replicates and dashes (–) indicating the nodes not represented in the 50% majority rule bootstrap consensus tree. The figure was generated using FigTree v1.4.3 (http://tree.bio.ed.ac.uk/software/figtree/).

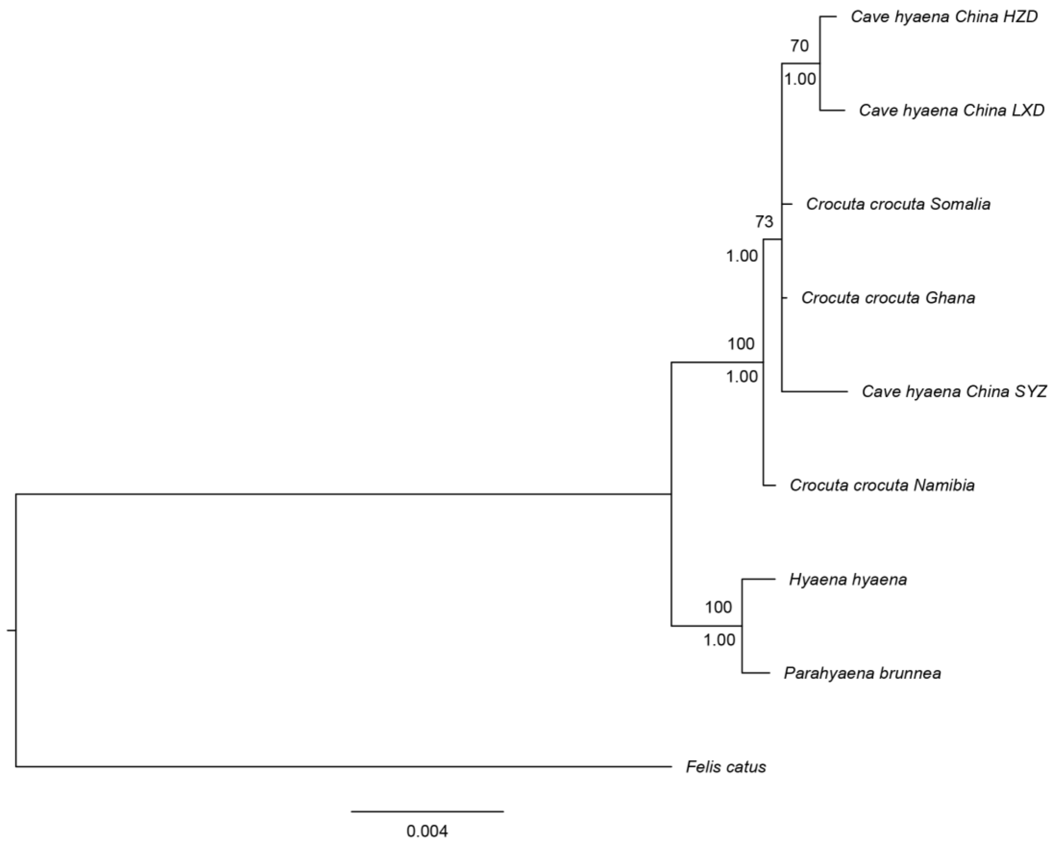

Figure S4. Fifty percent majority rule Bayesian consensus tree based on proteomic dataset 2, with *Felis catus* (domestic cat) as an outgroup. Scale bar represents branch length in units of substitutions per site. Values below nodes are Bayesian posterior probabilities for the descendant clade. The parsimony results are also attached on the consensus tree, with values above nodes indicating the percent bootstrap support generated from 1,000 bootstrap replicates. The figure was generated using FigTree v1.4.3 (http://tree.bio.ed.ac.uk/software/figtree/).

The phylogenetic tree generated from dataset 1 agrees well with the commonly accepted high level Feliformia classification based on morphological and genetic data (Fig. S3) [9-11]. The sequences from family Hyaenidae form a monophyletic group which is sister to all the other Feliformia species from family Felidae. Within the Felidae clade, the subfamily Pantherinae (big cats) is clearly differentiated from subfamily Felinae (small cats). As to the Hyaenidae clade, *Hyaena* and *Parahyaena* form a group, which is sister to all the modern spotted and cave hyenas. The phylogenetic tree based on dataset 2 has the same topology in the Hyaenidae clade as that of dataset 1, but with higher support values (BS and PP), perhaps due to more amino acid residues included in dataset 2 (Fig. S4).

Additional phylogenetic analyses

To address the importance of certain variable site (position 1449, COL1A2), additional phylogenetic analyses were performed based on a modified dataset from dataset 2. With all the Hyaenidae sequences called as X at position 1449, the results of parsimony and Bayesian analyses are displayed in Fig. S5 and S6. In the *Crocuta* clade, both the African

modern spotted hyenas and the east Asian cave hyenas could still be separated into two groups respectively. But the Namibian individual did not form a basal lineage anymore. In Fig. S6, the SYZ sample grouped together with the northern African spotted hyenas, but with a lower support (PP 0.31), which was sister to the Namibian individual (PP 0.42). The divergence time between the SYZ and the northern African individuals was estimated to be 1.01 Ma, which agreed well with that estimated from the original dataset 2. Compared with the time-scaled maximum clade credibility tree generated from the original dataset 2 (Fig. 4), here the placement of the Namibian individual was different. The different results between the original and modified dataset 2 indicated that the variable site (position 1449, COL1A2) has significant importance to distinguish different clades within the genus *Crocuta* based on ancient protein evidence, and to further support the gene flow between the Northern African spotted hyenas and east Asian cave hyenas. However, it is noteworthy that the frequency distribution of different amino acid types (I, V, possibly other types detected afterwards at position 1449) within Hyaenidae populations remains unclear yet due to limited number of whole genomes available so far. With more samples analyzed in the future, the phylogenetic relationships between modern spotted hyenas and east Asian cave hyenas could be confirmed or modified.

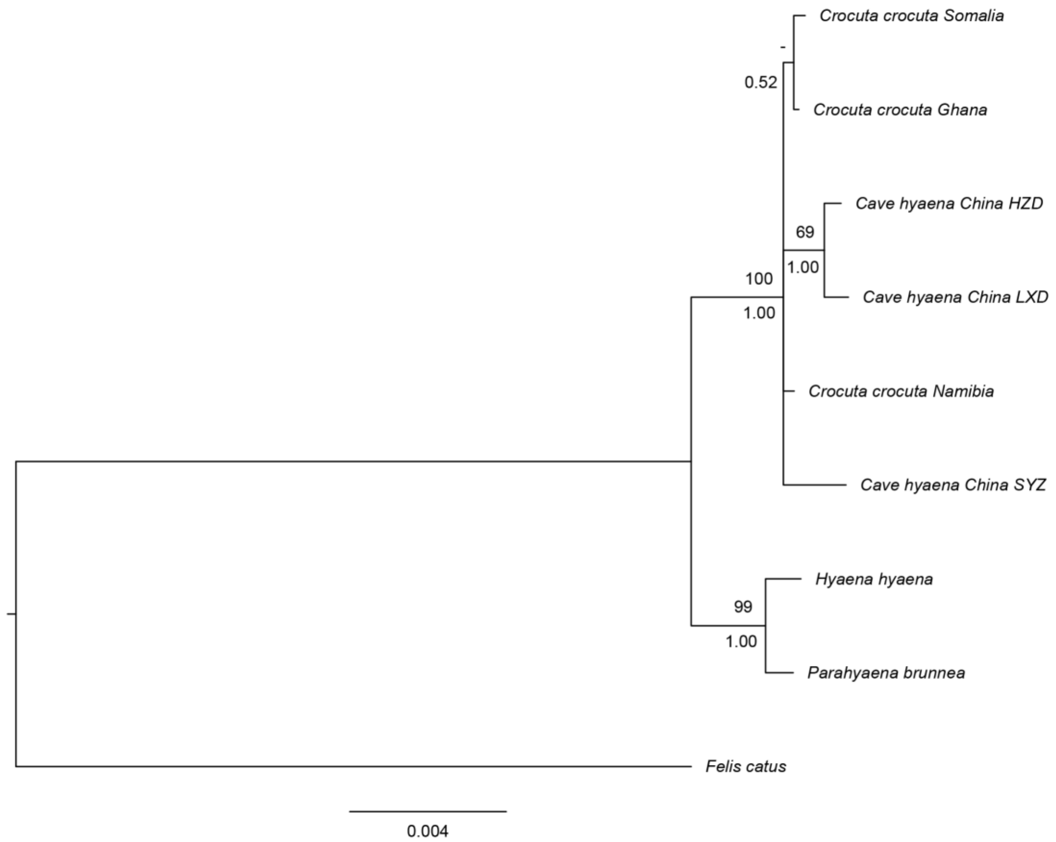

Figure S5. Fifty percent majority rule Bayesian consensus tree based on modified proteomic dataset 2 (all the Hyaenidae sequences called as X at position 1449), with *Felis catus* (domestic cat) as an outgroup. Scale bar represents branch length in units of substitutions per site. Values below nodes are Bayesian posterior probabilities for the descendant clade. The parsimony results are also attached on the consensus tree, with

values above nodes indicating the percent bootstrap support generated from 1,000 bootstrap replicates and dashes (–) indicating the nodes not represented in the 50% majority rule bootstrap consensus tree. The figure was generated using FigTree v1.4.3 (http://tree.bio.ed.ac.uk/software/figtree/).

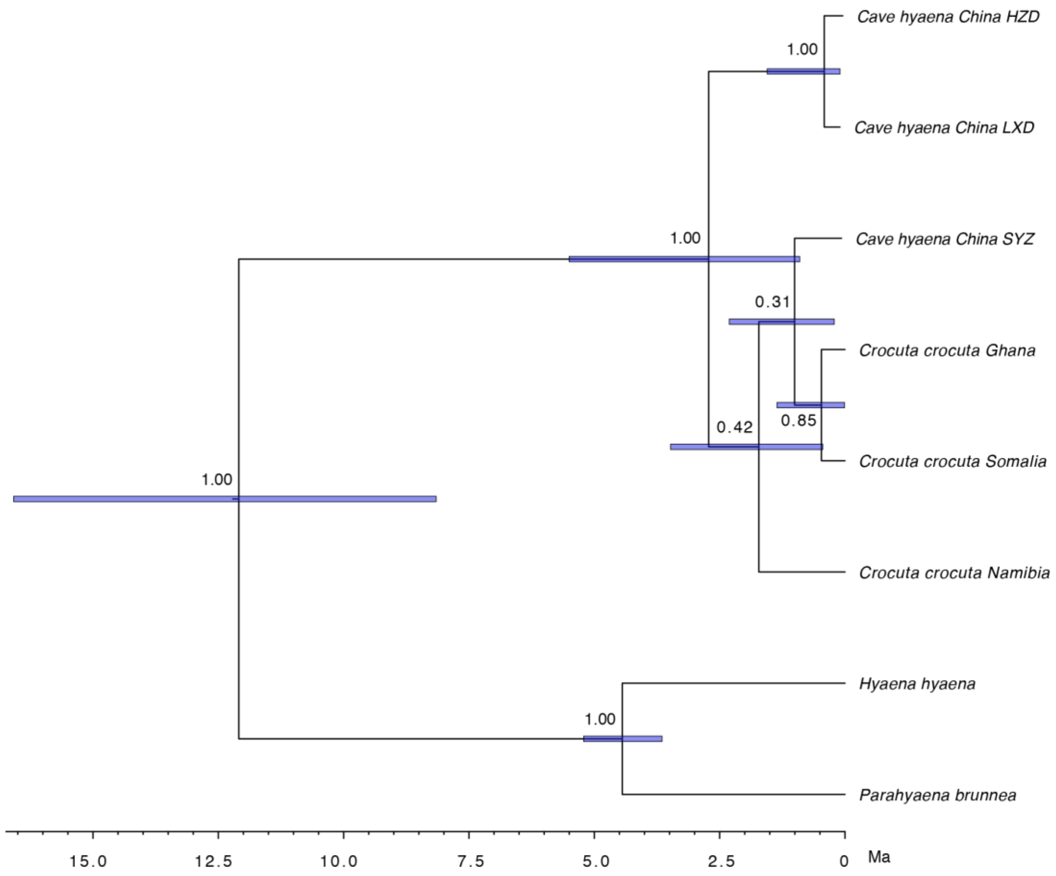

Figure S6. Time-scaled maximum clade credibility tree estimated using BEAST and a concatenated alignment of 10 proteins from 8 extant and extinct taxa (all the Hyaenidae sequences called as X at position 1449). Posterior Bayesian probabilities are indicated at nodes with a probability of ≤ 1. Horizontal error bars at each node represent 95% highest posterior density intervals. The figure was generated using FigTree v1.4.3 (http://tree.bio.ed.ac.uk/software/figtree/).

In an attempt to determine the relationships between our samples and other Eurasian cave hyenas, we incorporated the translated protein sequences from 11 low coverage genomes (1.6 - 6.3x) of 4 cave hyenas and 7 modern spotted hyenas [6]. The phylogenies based on the new dataset are displayed in Figs. S7 and S8. Even though the maximum clade credibility tree (Fig. S8, obtained from BEAST analysis) shows that the European cave hyenas form the basal lineages to other *Crocuta* individuals, the supports are low (just around 0.50 or lower). Actually, most of the clades in Fig. S8 have a posterior probability of below 0.5. Combining with the 50% majority rule consensus trees obtained from parsimony and Bayesian (carried out in Mrbayes) analyses (Fig. S7), the relationships within most of the cave hyenas and modern spotted hyenas could not be resolved. What is more, the node age of the *Crocuta* clade is estimated to be much older

than that estimated from nuclear genome data. The inconclusive phylogeny and the older age could be caused by the addition of substitutions brought about by sequencing error or DNA damage, which is more prone to be exposed in low coverage genomes. To obtain clearer and more accurate evolutionary results of cave hyenas, more reliable data should be chosen for the phylogenetic analysis.

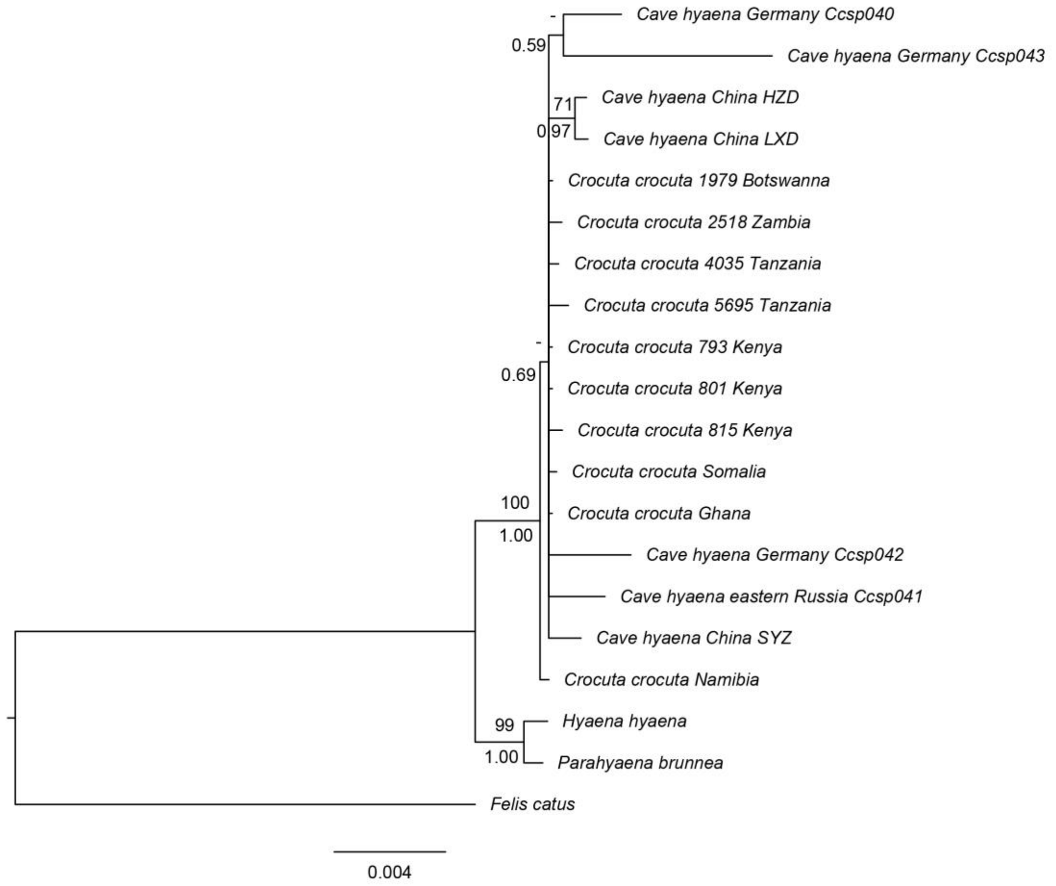

Figure S7. Fifty percent majority rule Bayesian consensus tree using a concatenated alignment of 10 proteins from 20 extant and extinct taxa, with *Felis catus* (domestic cat) as an outgroup. Scale bar represents branch length in units of substitutions per site. Values below nodes are Bayesian posterior probabilities for the descendant clade. The parsimony results are also attached on the consensus tree, with values above nodes indicating the percent bootstrap support generated from 1,000 bootstrap replicates and dashes (–) indicating the nodes not represented in the 50% majority rule bootstrap consensus tree. The figure was generated using FigTree v1.4.3 (http://tree.bio.ed.ac.uk/software/figtree/).

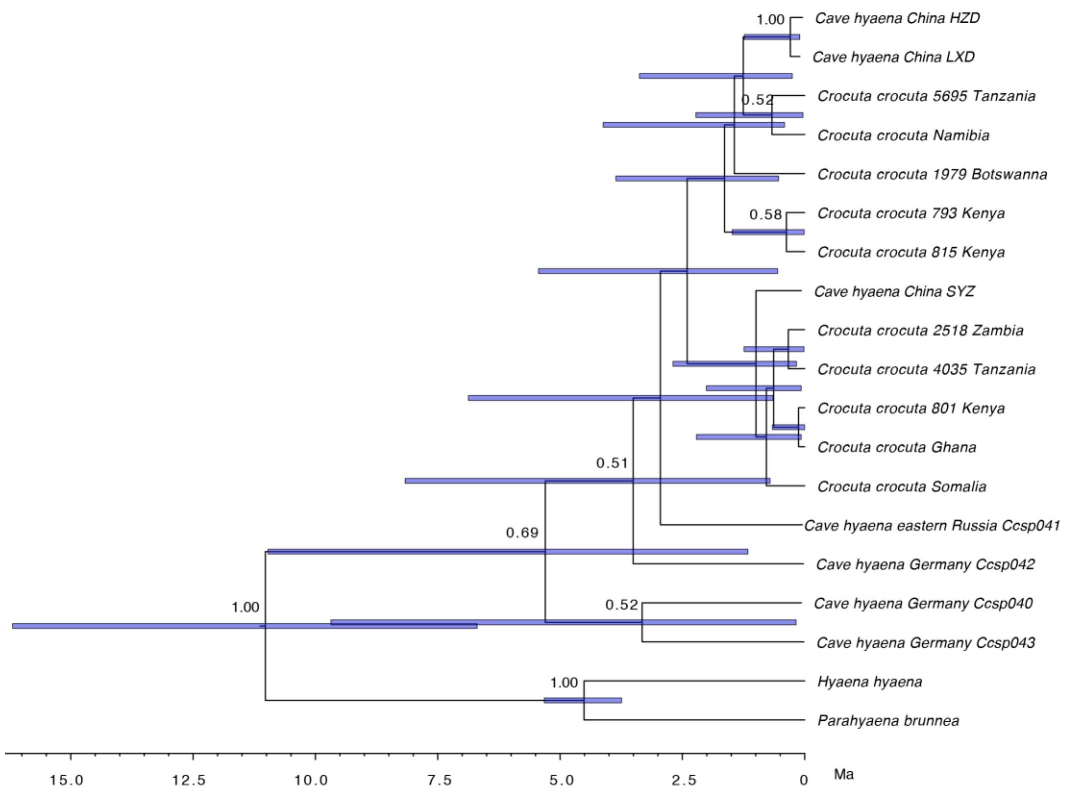

Figure S8. Time-scaled maximum clade credibility tree estimated using BEAST and a concatenated alignment of 10 proteins from 19 extant and extinct taxa. Posterior Bayesian probabilities are indicated at nodes with a probability of ≤ 1. Only the posterior probabilities of above 0.5 are shown. Horizontal error bars at each node represent 95% highest posterior density intervals. The figure was generated using FigTree v1.4.3 (http://tree.bio.ed.ac.uk/software/figtree/).

All the details of phylogenetic analyses carried out in BEAST and Mrbayes were provided in XML and NEX files, which were also uploaded as supplementary materials (XML Files S1-3 and NEX Files S1-4).

The spectra generated from prescreening analyses and the ion match tables for the MS/MS spectra used in Figs. S1 and S2

Figures S9-14 show the FTIR spectra of the cave hyena samples. Figures S15-17 show the MALDI-TOF spectra of three cave hyena (sub)samples. The spectra of the other three fossil subsamples (LXD-1D, LXD-2B, and LXD-2D) were not displayed because of their poor signal. The peptide markers (P1-G) [4,12] were annotated beside their related mass. Figures S18-23 show the ion match tables for the example MS/MS spectra used in Figs. S1 and S2.

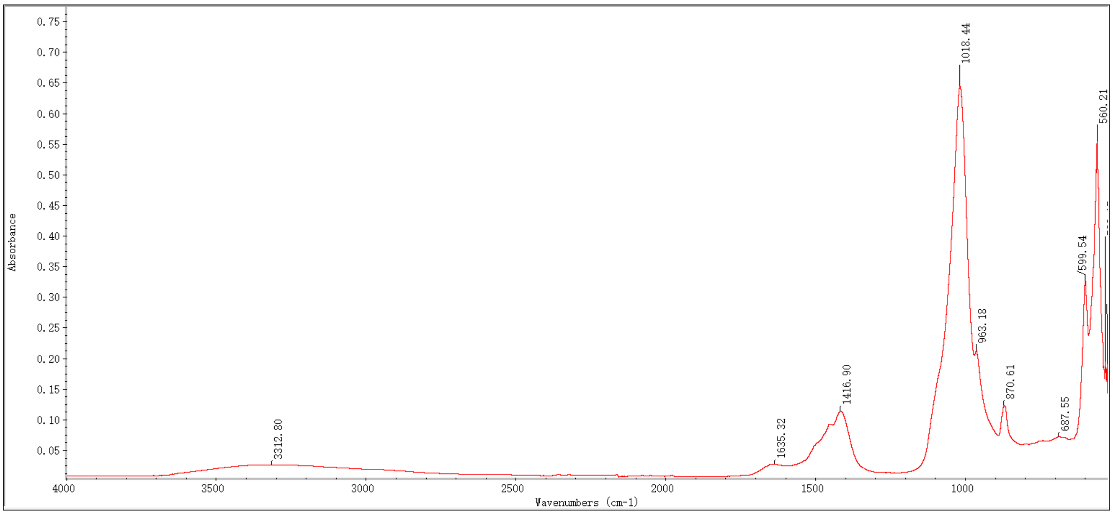

Figure S9. The FTIR spectrum of the SYZ sample.

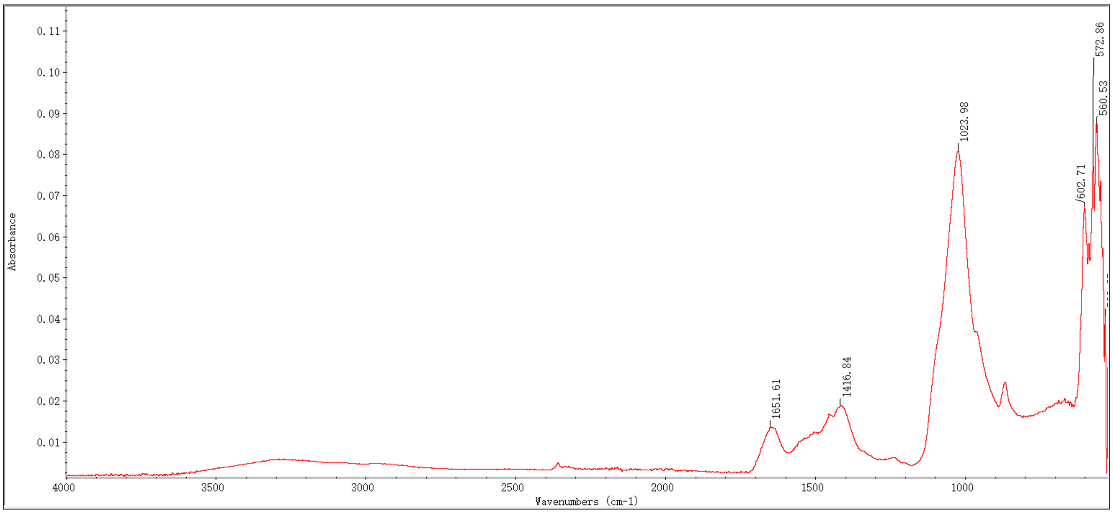

Figure S10. The FTIR spectrum of the HZD sample.

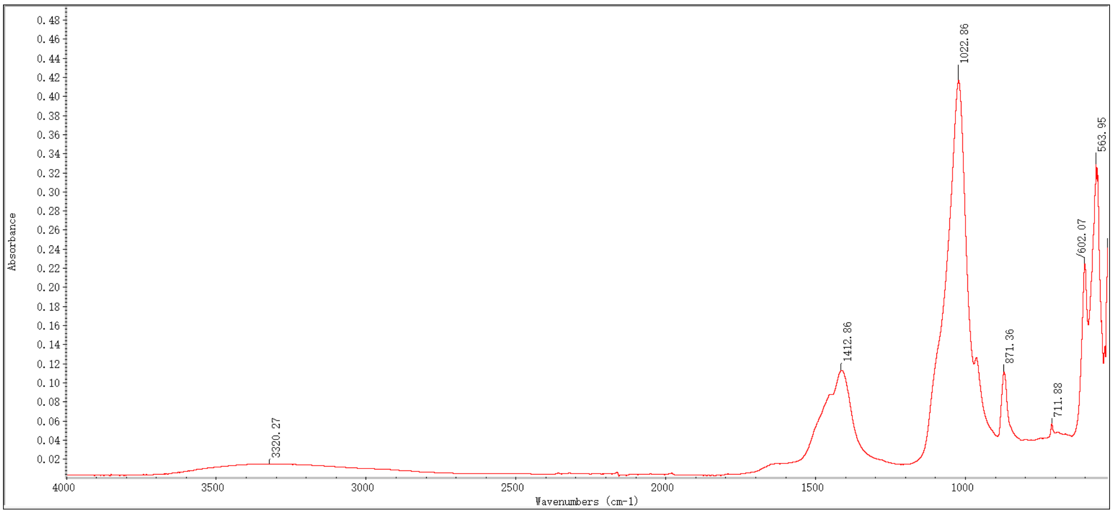

Figure S11. The FTIR spectrum of the LXD-1B subsample.

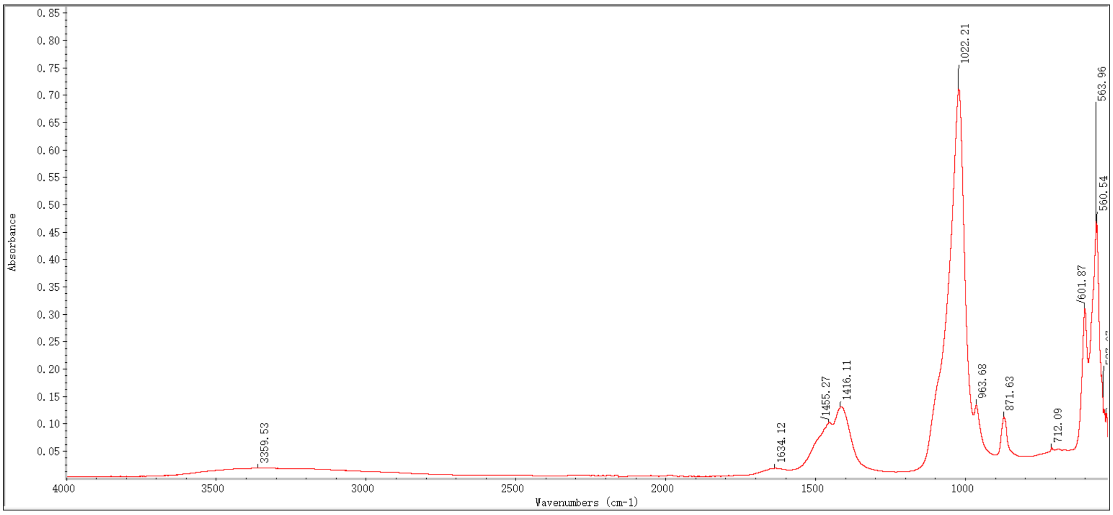

Figure S12. The FTIR spectrum of the LXD-1D subsample.

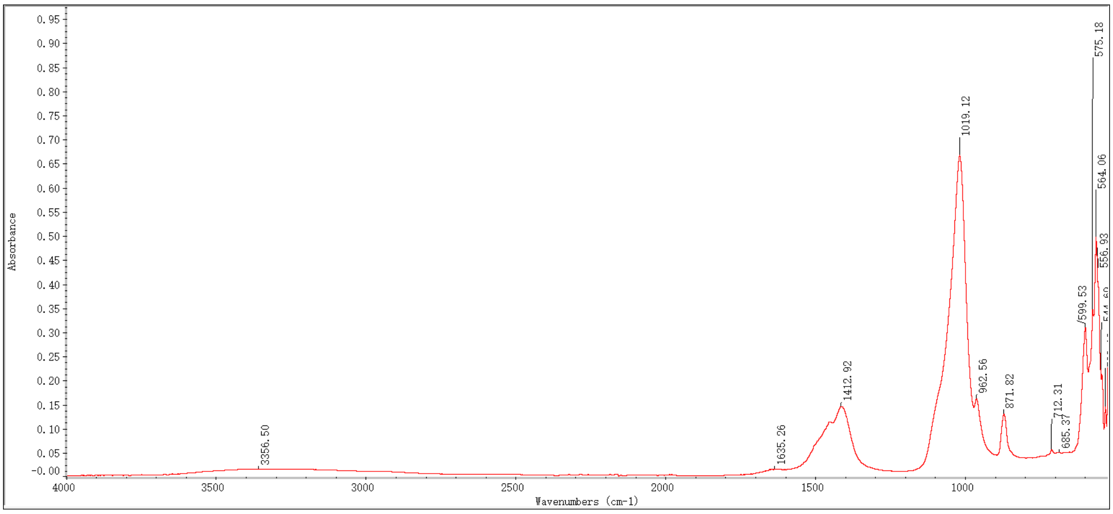

Figure S13. The FTIR spectrum of the LXD-2B subsample.

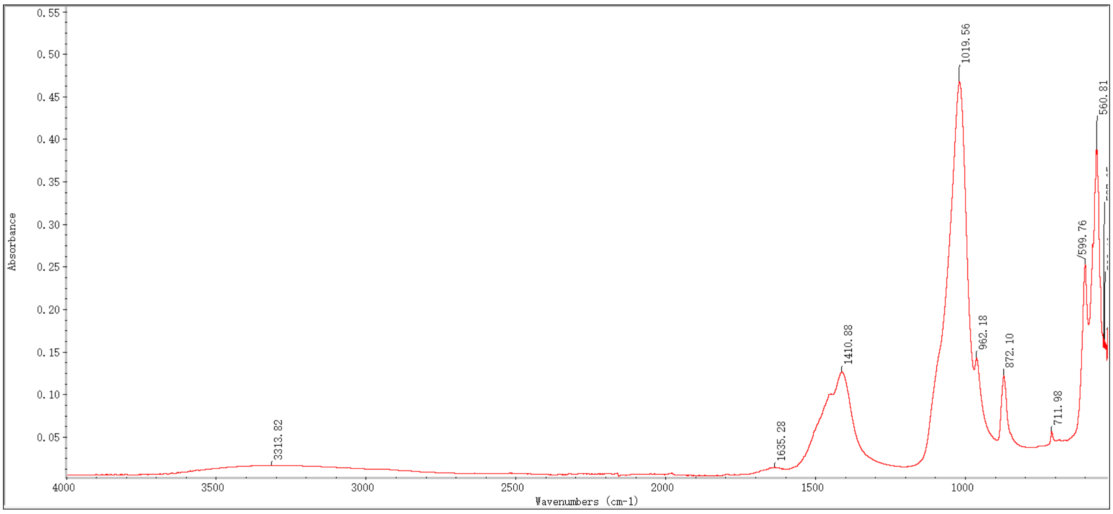

Figure S14. The FTIR spectrum of the LXD-2D subsample.

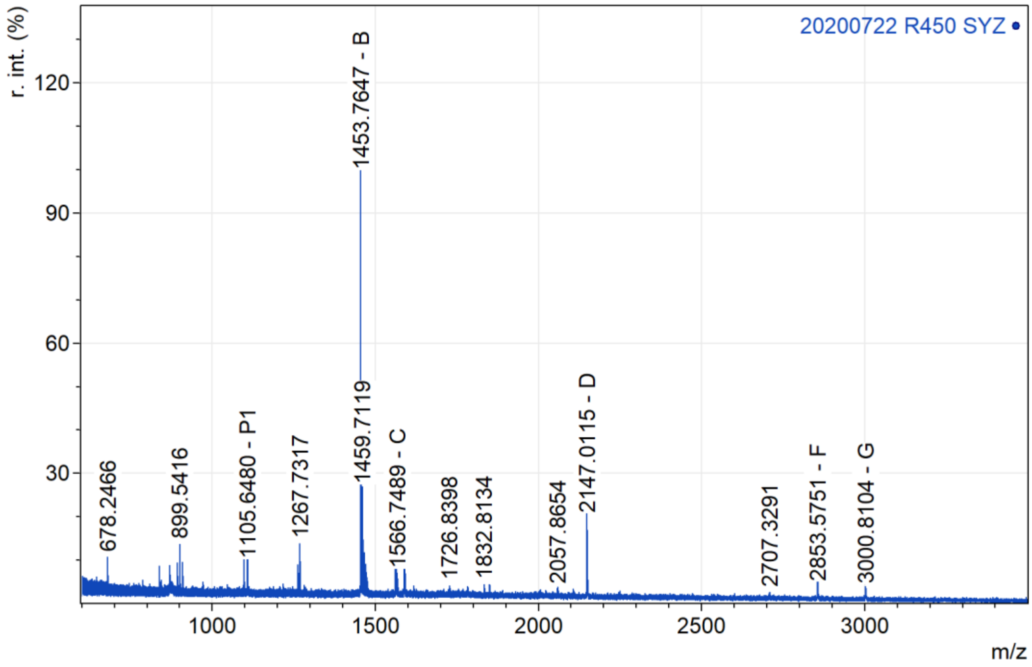

Figure S15. The MALDI-TOF spectrum of the SYZ sample.

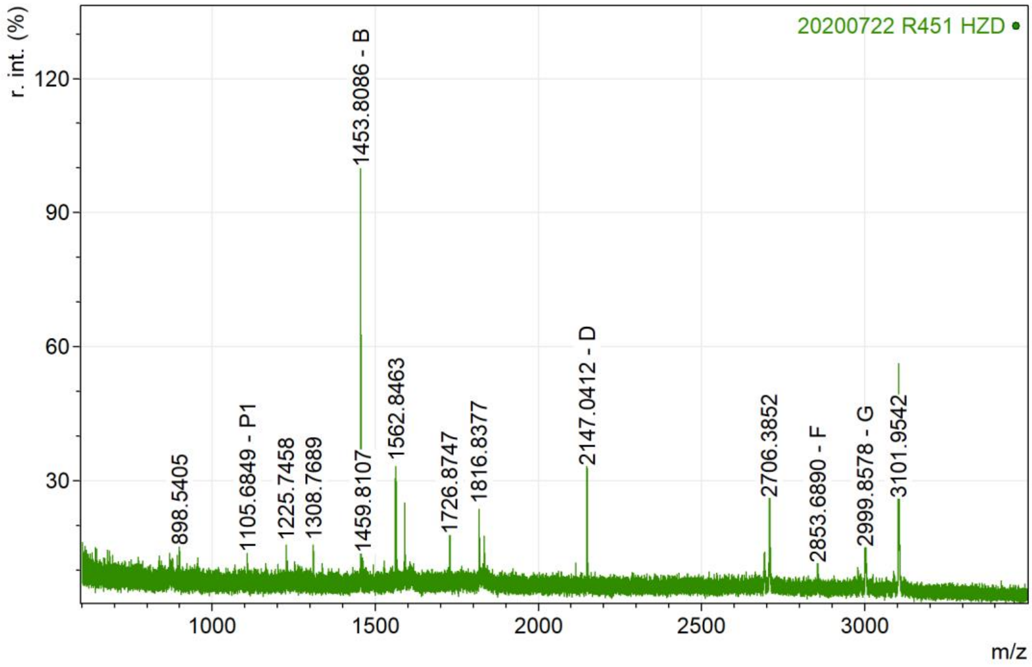

Figure S16. The MALDI-TOF spectrum of the HZD sample.

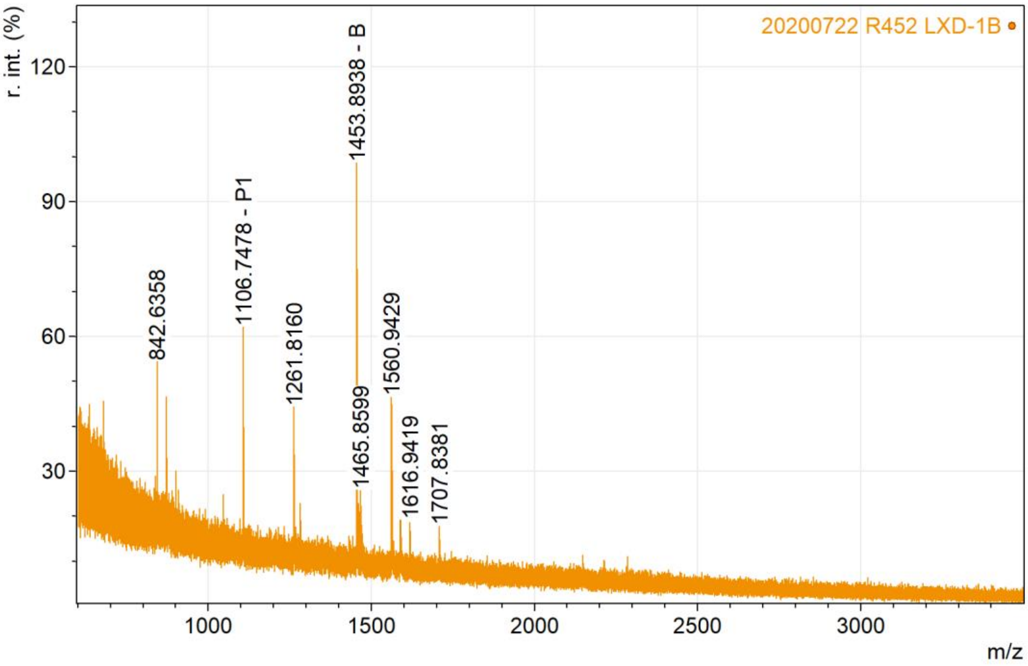

Figure S17. The MALDI-TOF spectrum of the LXD-1B subsample.

#	b	b-H2O	b-NH3	b (2+)	Seq	y	y-H2O	y-NH3	y (2+)	#
1	130.05	112.09	113.07	65.53	E					12
2	187.07	169.06	170.04	94.04	G	1087.52	1069.51	1070.49	544.26	11
3	284.16	266.11	267.10	142.56	P	1030.50	1012.49	1013.47	515.75	10
4	431.16	413.15	414.13	216.08	M(+15.99)	933.44	915.44	916.42	467.20	9
5	488.18	470.17	471.15	244.59	G	786.41	768.40	769.39	393.71	8
6	587.25	569.23	570.22	294.12	V	729.39	711.38	712.36	365.22	7
7	700.30	682.29	683.27	350.65	P(+15.99)	630.32	612.31	613.30	315.66	6
8	757.32	739.31	740.29	379.16	G	517.27	499.26	500.25	259.14	5
9	870.45	852.39	853.38	435.70	I	460.25	442.24	443.22	230.63	4
10	985.43	967.42	968.40	493.22	D	347.17	329.16	330.14	174.08	3
11	1042.45	1024.44	1025.42	521.73	G	232.14	214.13	215.14	116.57	2
12					R	175.12	157.11	158.09	88.06	1

Figure S18. The ion match table for the example MS/MS spectrum used in Fig. S1a.

#	b	b-H2O	b-NH3	b (2+)	Seq	y	y-H2O	y-NH3	y (2+)	#
1	130.05	112.04	113.02	65.53	E					22
2	187.07	169.06	170.04	94.04	G	1977.00	1958.99	1959.97	989.00	21
3	284.16	266.11	267.10	142.56	P	1919.97	1901.96	1902.95	960.49	20
4	431.16	413.15	414.13	216.08	M(+15.99)	1822.91	1804.91	1805.90	911.96	19
5	488.18	470.17	471.17	244.59	G	1675.88	1657.88	1658.86	838.44	18
6	587.25	569.24	570.22	294.12	V	1618.86	1600.86	1601.84	809.93	17
7	700.30	682.29	683.27	350.65	P(+15.99)	1519.79	1501.79	1502.78	760.40	16
8	757.32	739.31	740.29	379.18	G	1406.76	1388.74	1389.72	703.87	15
9	870.40	852.39	853.38	435.70	I	1349.71	1331.72	1332.70	675.36	14
10	985.43	967.42	968.40	493.22	D	1236.64	1218.63	1219.62	618.82	13
11	1042.45	1024.44	1025.42	521.73	G	1121.62	1103.60	1104.58	561.31	12
12	1198.55	1180.54	1181.53	599.78	R	1064.60	1046.59	1047.57	532.80	11
13	1311.60	1293.59	1294.57	656.30	P(+15.99)	908.49	890.48	891.47	454.75	10
14	1368.62	1350.61	1351.59	684.81	G	795.44	777.44	778.42	398.22	9
15	1465.67	1447.66	1448.65	733.34	P	738.41	720.42	721.40	369.72	8
16	1578.76	1560.75	1561.73	789.88	I	641.33	623.36	624.35	321.16	7
17	1635.78	1617.77	1618.75	818.39	G	528.29	510.28	511.26	264.64	6
18	1732.83	1714.82	1715.81	866.92	P	471.27	453.26	454.24	236.13	5
19	1803.87	1785.86	1786.84	902.43	A	374.21	356.20	357.19	187.61	4
20	1860.89	1842.88	1843.86	930.95	G	303.18	285.17	286.14	152.09	3
21	1931.93	1913.92	1914.90	966.46	A	246.16	228.15	229.13	123.58	2
22					R	175.12	157.11	158.09	88.06	1

Figure S19. The ion match table for the example MS/MS spectrum used in Fig. S1b.

#	b	b-H2O	b-NH3	b (2+)	Seq	y	y-H2O	y-NH3	y (2+)	#
1	130.05	112.04	113.02	65.53	E					22
2	187.07	169.06	170.04	94.04	G	1977.00	1958.99	1959.97	989.00	21
3	284.12	266.11	267.12	142.56	P	1919.98	1901.96	1902.95	960.49	20
4	431.16	413.15	414.13	216.08	M(+15.99)	1822.92	1804.91	1805.90	911.96	19
5	488.18	470.17	471.17	244.59	G	1675.88	1657.88	1658.86	838.44	18
6	587.25	569.24	570.22	294.12	V	1618.86	1600.86	1601.84	809.93	17
7	700.30	682.29	683.27	350.65	P(+15.99)	1519.79	1501.78	1502.78	760.40	16
8	757.32	739.31	740.29	379.16	G	1406.76	1388.74	1389.72	703.87	15
9	870.40	852.39	853.38	435.70	I	1349.71	1331.72	1332.70	675.36	14
10	985.43	967.42	968.40	493.22	D	1236.64	1218.63	1219.61	618.82	13
11	1042.45	1024.44	1025.42	521.73	G	1121.62	1103.61	1104.58	561.31	12
12	1198.55	1180.54	1181.53	599.78	R	1064.60	1046.59	1047.57	532.80	11
13	1311.60	1293.59	1294.57	656.30	P(+15.99)	908.49	890.48	891.47	454.75	10
14	1368.62	1350.61	1351.59	684.81	G	795.45	777.44	778.42	398.22	9
15	1465.67	1447.66	1448.65	733.34	P	738.43	720.42	721.40	369.71	8
16	1578.76	1560.75	1561.73	789.88	I	641.33	623.36	624.35	321.16	7
17	1635.78	1617.77	1618.75	818.39	G	528.29	510.28	511.26	264.64	6
18	1732.83	1714.82	1715.81	866.92	P	471.26	453.26	454.24	236.13	5
19	1803.87	1785.86	1786.84	902.43	A	374.21	356.20	357.19	187.61	4
20	1860.89	1842.88	1843.86	930.95	G	303.18	285.17	286.14	152.09	3
21	1931.93	1913.92	1914.90	966.46	A	246.16	228.15	229.13	123.58	2
22					R	175.12	157.10	158.09	88.06	1

Figure S20. The ion match table for the example MS/MS spectrum used in Fig. S1c.

#	b	b-H2O	b-NH3	b (2+)	Seq	y	y-H2O	y-NH3	y (2+)	#
1	102. 06	84. 04	85. 03	51. 53	T					13
2	159. 08	141. 10	142. 05	80. 04	G	1084. 53	1066. 53	1067. 51	542. 77	12
3	274. 10	256. 09	257. 12	137. 55	D	1027. 51	1009. 51	1010. 49	514. 26	11
4	387. 15	369. 14	370. 12	194. 08	P(+15. 99)	912. 49	894. 47	895. 48	456. 74	10
5	444. 17	426. 16	427. 15	222. 59	G	799. 44	781. 43	782. 42	400. 22	9
6	531. 20	513. 19	514. 18	266. 10	S	742. 42	724. 41	725. 41	371. 71	8
7	630. 27	612. 26	613. 25	315. 64	V	655. 39	637. 38	638. 37	328. 19	7
8	687. 29	669. 28	670. 27	344. 15	G	556. 32	538. 31	539. 30	278. 66	6
9	784. 35	766. 34	767. 32	392. 67	P	499. 30	481. 28	482. 27	250. 15	5
10	855. 38	837. 37	838. 36	428. 19	A	402. 24	384. 24	385. 22	201. 62	4
11	912. 41	894. 40	895. 38	456. 70	G	331. 21	313. 20	314. 18	166. 10	3
12	1011. 47	993. 46	994. 45	506. 24	V	274. 19	256. 18	257. 12	137. 59	2
13					R	175. 12	157. 11	158. 09	88. 06	1

Figure S21. The ion match table for the example MS/MS spectrum used in Fig. S2a.

#	b	b-H2O	b-NH3	b (2+)	Seq	y	y-H2O	y-NH3	y (2+)	#
1	58. 03	40. 02	41. 00	29. 51	G					18
2	205. 10	187. 07	188. 10	103. 05	F	1666. 83	1648. 82	1649. 80	833. 91	17
3	318. 15	300. 12	301. 15	159. 57	P(+15. 99)	1519. 76	1501. 73	1502. 73	760. 38	16
4	375. 17	357. 14	358. 17	188. 10	G	1406. 69	1388. 71	1389. 69	703. 86	15
5	462. 20	444. 19	445. 17	231. 60	S	1349. 65	1331. 68	1332. 66	675. 30	14
6	618. 28	600. 27	601. 27	309. 65	R	1262. 66	1244. 65	1245. 63	631. 83	13
7	675. 30	657. 30	658. 29	338. 16	G	1106. 55	1088. 52	1089. 53	553. 78	12
8	774. 37	756. 35	757. 36	387. 69	V	1049. 53	1031. 53	1032. 51	525. 23	11
9	903. 42	885. 42	886. 41	452. 22	Q(+. 98)	950. 46	932. 45	933. 45	475. 73	10
10	960. 45	942. 43	943. 43	480. 73	G	821. 42	803. 42	804. 40	411. 21	9
11	1057. 51	1039. 50	1040. 48	529. 25	P	764. 40	746. 37	747. 38	382. 70	8
12	1170. 55	1152. 54	1153. 53	585. 78	P(+15. 99)	667. 35	649. 34	650. 33	334. 18	7
13	1227. 58	1209. 57	1210. 55	614. 29	G	554. 30	536. 29	537. 28	277. 65	6
14	1324. 63	1306. 62	1307. 60	662. 81	P	497. 28	479. 27	480. 26	249. 14	5
15	1395. 67	1377. 66	1378. 64	698. 33	A	400. 22	382. 22	383. 15	200. 62	4
16	1452. 69	1434. 68	1435. 66	726. 84	G	329. 19	311. 18	312. 17	165. 10	3
17	1549. 74	1531. 73	1532. 71	775. 37	P	272. 12	254. 11	255. 14	136. 59	2
18					R	175. 12	157. 10	158. 09	88. 06	1

Figure S22. The ion match table for the example MS/MS spectrum used in Fig. S2b.

#	b	b-H20	b-NH3	b (2+)	Seq	y	y-H20	y-NH3	y (2+)	#
1	58.03	40.02	41.00	29.51	G					18
2	205.10	187.07	188.10	103.05	F	1666.83	1648.82	1649.80	833.91	17
3	318.15	300.12	301.15	159.57	P(+15.99)	1519.74	1501.73	1502.73	760.38	16
4	375.17	357.16	358.14	188.10	G	1406.69	1388.70	1389.69	703.86	15
5	462.20	444.19	445.17	231.60	S	1349.69	1331.68	1332.66	675.32	14
6	618.29	600.26	601.27	309.65	R	1262.66	1244.65	1245.63	631.83	13
7	675.32	657.30	658.29	338.16	G	1106.56	1088.55	1089.55	553.78	12
8	774.37	756.35	757.36	387.69	V	1049.53	1031.53	1032.51	525.27	11
9	903.40	885.42	886.41	452.22	Q(+.98)	950.47	932.46	933.44	475.73	10
10	960.43	942.44	943.43	480.73	G	821.42	803.42	804.40	411.21	9
11	1057.51	1039.50	1040.48	529.25	P	764.40	746.39	747.38	382.70	8
12	1170.55	1152.54	1153.53	585.78	P(+15.99)	667.35	649.34	650.33	334.18	7
13	1227.58	1209.57	1210.55	614.29	G	554.30	536.29	537.28	277.65	6
14	1324.63	1306.62	1307.60	662.81	P	497.28	479.27	480.26	249.14	5
15	1395.67	1377.66	1378.64	698.33	A	400.22	382.22	383.20	200.62	4
16	1452.69	1434.68	1435.66	726.84	G	329.19	311.18	312.17	165.10	3
17	1549.74	1531.73	1532.71	775.37	P	272.12	254.16	255.14	136.59	2
18					R	175.12	157.10	158.09	88.06	1

Figure S23. The ion match table for the example MS/MS spectrum used in Fig. S2c.

Legends for supplementary tables and other supplementary Files

Table S1. The identified proteins in the SYZ sample.

Table S2. The identified proteins in the HZD sample.

Table S3. The identified proteins in the LXD sample.

Table S4. Comparative datasets used for phylogenetic reconstructions, referencing database sources (with accession numbers) or associated genome/proteome publications.

Fasta File S1. Comparative dataset 1 alignment used for phylogenetic reconstructions, where COL1 A1 and COL1A2 were concatenated, with COL1A1 ranging from position 1 to position 1057, and COL1A2 from 1058 to 2098.

Fasta File S2. Comparative dataset 2 alignment used for phylogenetic reconstructions, where the proteins were concatenated in the following order:

COL1A1 = 1-1057;

COL1A2 = 1058-2098;

AHSG = 2099-2462;

CHAD = 2463-2821;

OMD = 2822-3237;

SPARC = 3238-3540;

VTN = 3541-4012;

BGN = 4013-4384;

CLEC3B = 4385-4586;

SERPINF1 = 4587-5003.

XML File S1. The details of phylogenetic analysis carried out in BEAST using a concatenated alignment of 10 proteins from 8 extant and extinct taxa.

XML File S2. The details of phylogenetic analysis carried out in BEAST using a concatenated alignment of 10 proteins from 8 extant and extinct taxa. All the Hyaenidae sequences were called as X at position 1449.

XML File S3. The details of phylogenetic analysis carried out in BEAST using a concatenated alignment of 10 proteins from 19 extant and extinct taxa.

NEX File S1. The details of phylogenetic analysis carried out in Mrbayes using proteomic dataset 1, with *Ailuropoda melanoleuca* (giant panda) as an outgroup.

NEX File S2. The details of phylogenetic analysis carried out in Mrbayes using proteomic dataset 2, with *Felis catus* (domestic cat) as an outgroup.

NEX File S3. The details of phylogenetic analysis carried out in Mrbayes using a modified dataset 2, with *Felis catus* (domestic cat) as an outgroup. All the Hyaenidae sequences were called as X at position 1449.

NEX File S4. The details of phylogenetic analysis carried out in Mrbayes using a concatenated alignment of 10 proteins from 20 extant and extinct taxa, with *Felis catus* (domestic cat) as an outgroup.

Extended Data File S1. The raw data of all the MALDI-TOF MS spectra.

References

1 Welker, F. Palaeoproteomics for human evolution studies. *Quaternary Science Reviews* **190**, 137-147, doi:https://doi.org/10.1016/j.quascirev.2018.04.033 (2018).

2 Buckley, M. & Wadsworth, C. Proteome degradation in ancient bone; what ancient proteins can tell us. *Palaeogeography, Palaeoclimatology, Palaeoecology* **416**, 69-79, doi:http://dx.doi.org/10.1016/j.palaeo.2014.06.026 (2014).

3 Welker, F. *et al.* Ancient proteins resolve the evolutionary history of Darwin/'s South American ungulates. *Nature* **522**, 81-84 (2015).

4 Welker, F. *et al.* Palaeoproteomic evidence identifies archaic hominins associated with the Châtelperronian at the Grotte du Renne. *Proceedings of the National Academy of Sciences* **113**, 11162-11167 (2016).

5 Westbury, M. V. *et al.* Extended and Continuous Decline in Effective Population Size Results in Low Genomic Diversity in the World's Rarest Hyena Species, the Brown Hyena. *Molecular Biology and Evolution* **35**, 1225-1237, doi:10.1093/molbev/msy037 (2018).

6 Westbury, M. V. *et al.* Hyena paleogenomes reveal a complex evolutionary history of cross-continental gene flow between spotted and cave hyena. *Science Advances* **6**, eaay0456, doi:10.1126/sciadv.aay0456 (2020).

7 Presslee, S. *et al.* Palaeoproteomics resolves sloth relationships. *Nature Ecology & Evolution* **3**, 1121–1130, doi:10.1038/s41559-019-0909-z (2019).

8 Welker, F. Elucidation of cross-species proteomic effects in human and hominin bone proteome identification through a bioinformatics experiment. *BMC Evolutionary Biology* **18**, 23 (2018).

9 Koepfli, K.-P. *et al.* Molecular systematics of the Hyaenidae: Relationships of a relictual lineage resolved by a molecular supermatrix. *Molecular Phylogenetics and Evolution* **38**, 603-620, doi:https://doi.org/10.1016/j.ympev.2005.10.017 (2006).

10 Johnson, W. E. *et al.* The Late Miocene Radiation of Modern Felidae: A Genetic Assessment. *Science* **311**, 73-77, doi:10.1126/science.1122277 (2006).

11 Werdelin, L., Yamaguchi, N., Johnson, W. E. & O'Brien, S. J. Phylogeny and evolution of cats (Felidae). *Biology and Conservation of Wild Felids* **12**, 59-82 (2010).

12 Buckley, M., Collins, M., Thomas-Oates, J. & Wilson, J. C. Species identification by analysis of bone collagen using matrix-assisted laser desorption/ionisation time-of-flight mass spectrometry. *Rapid Communications in Mass Spectrometry* **23**, 3843-3854, doi:10.1002/rcm.4316 (2009).
